# Identifying Tipping Points during Healthy Brain Aging through Single‐Nucleus Transcriptomic Analysis

**DOI:** 10.1002/advs.202505779

**Published:** 2025-08-19

**Authors:** Peiru Wu, Xuyu Zhao, Zixin Chen, Jingying Huang, Tengteng Dai, Jianxin Zhou, Luyao Xiao, Luonan Chen, Robert Chunhua Zhao, Jiao Wang

**Affiliations:** ^1^ School of Life Sciences Shanghai University Shanghai 200444 China; ^2^ School of Mathematical Sciences and School of AI Shanghai Jiao Tong University Shanghai 200230 China; ^3^ Centre of Excellence in Tissue Engineering Chinese Academy of Medical Sciences Beijing 100005 China; ^4^ Beijing Key Laboratory of New Drug Development and Clinical Trial of Stem Cell Therapy Beijing 100005 China; ^5^ Institute of Basic Medical Sciences Chinese Academy of Medical Sciences School of Basic Medicine Peking Union Medical College Beijing 100005 China; ^6^ School of Life Science and Technology Shandong Vocational University of Foreign Affairs Jinan 250131 China

**Keywords:** ageing, brain cells, dynamic network biomarkers, nonlinear, single‐cell RNA sequencing, tipping point

## Abstract

Brain aging significantly impairs cognitive and behavioral functions. While some nonlinear aging studies have identified age‐specific aging peaks at certain ages, the influence of different cell types on brain aging fluctuations across the lifespan remains unclear. This study, approaching from the interdisciplinary perspective of brain aging and systems dynamics, extends the nonlinear aging analysis to the cellular level, using single‐cell transcriptomic data to analyze 45 healthy elderly brain samples aged 29–94 years. Describing cellular and molecular differences in the aging process, neuron proportion is downregulated but relatively stable with low variability after aging, while glial cells are significantly upregulated and highly unstable. Notably, peaks in nonlinear molecular fluctuations are observed in aging at ages 60, 70, and 79. The nonlinear features prompted the introduction of a dynamic network biomarker and the identification of 56–60 years as the tipping point in the brain's healthy aging process, then suggesting that glia predominantly mediate this process and exploring the underlying features and mechanisms. This work investigates the tipping point of aging at single‐cell resolution and provides new research strategies for early diagnosis and intervention of aging‐related neurological diseases.

## Graphical abstract

This study applies single‐nucleus transcriptomics and systems dynamics to uncover nonlinear aging patterns in healthy human brains. By introducing dynamic network biomarker theory, the authors identify a critical tipping point at ages 56–60 during aging, and highlight glial instability as a key mediator. These findings provide new insights into brain aging and offer potential avenues for early intervention.

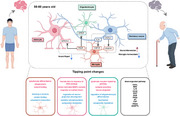

## 1 Introduction

As the population ages, aging‐related diseases such as Alzheimer's disease, Parkinson's disease, and mild cognitive impairment pose serious challenges to global healthcare.^[^ ^1^, ^2^, ^3^ ^]^ Aging is not only a major risk factor for a variety of neurological diseases but also a root cause of the decline of several physiological functions, especially brain aging.^[^ ^4^ ^]^ Brain aging, as an important biological process, affects many aspects of cognition, behavior, and emotion.^[^ ^5^, ^6^ ^]^ Studies have shown that brain aging not only involves nerve cell damage and death but also is closely related to neuroinflammation, synaptic degeneration, and damage to the blood–brain barrier.^[^ ^7^, ^8^, ^9^ ^]^ Therefore, studying the key mechanisms in the aging process and exploring the biomarkers of aging are the basis for revealing the relationship between aging and neurological diseases. In recent years, continuous studies have shown that organismal aging is characterized by nonlinear features,^[^ ^10^ ^]^ and Lehallier et al.^[^ ^11^ ^]^ developed a new bioinformatics method that reveals the waves of proteomic change at 34, 60, and 78 years of life in plasma proteome. Transitional periods of aging in the 30s and 50s were also identified in a study of the multimodal aging clock in women.^[^ ^12^ ^]^ A multiomics dataset of 108 participants successfully captured the dynamic and nonlinear molecular changes that occur around the ages of 40 and 60 during human aging.^[^ ^13^ ^]^ There is no exception in brain aging, and a recent study on proteins and the brain age gap, which constructed a model of aging from multimodal brain imaging data, detected the ebb and flow of the plasma proteome during brain aging and identified peaks in brain age‐related changes at ages 57, 70, and 78 years.^[^ ^14^ ^]^ While these studies provide crucial insights into the systemic and molecular dynamics of aging, they fall short of offering the cellular‐level resolution needed to unravel the full complexity and heterogeneity of the aging process. Cellular‐level investigations are indispensable, as individual cells within tissues and organs often follow unique aging trajectories that remain obscured in bulk or multimodal analyses.

Here, we collected and integrated single‐cell transcriptomic data published in recent years from human brain tissues aged 29 to 94 years to analyze the complex cell‐type composition and molecular changes during healthy aging, mainly including cortical and hippocampal regions that are highly correlated with aging and neurological diseases. A single‐cell transcriptome atlas of the human healthy aging brain of 1 million cells with quality control (QC) was ultimately constructed. We performed detailed senescence‐related analyses of different cell types and found that neurons are downregulated in senescence but exhibit have low variability and are relatively stable, whereas glial cells are significantly upregulated and highly unstable by senescence. In addition, we similarly observed nonlinear fluctuations during brain aging in single‐cell genomics, and combined this with computational methods for dynamic network biomarkers (DNBs) to identify age stage oriented to a tipping point of aging,^[^ ^15^ ^]^ and depict cellular and molecular features of this state, and also candidate new brain aging markers. Our results provide single‐cell omics evidence of the nonlinear features of brain aging and offer new strategies and targets for early diagnosis and personalized treatment of brain aging‐related diseases.

## 2 Results

### 2.1 Construction of a Single‐Cell Transcriptomic Atlas across Healthy Adult Brain Aging

**Figure 1:**
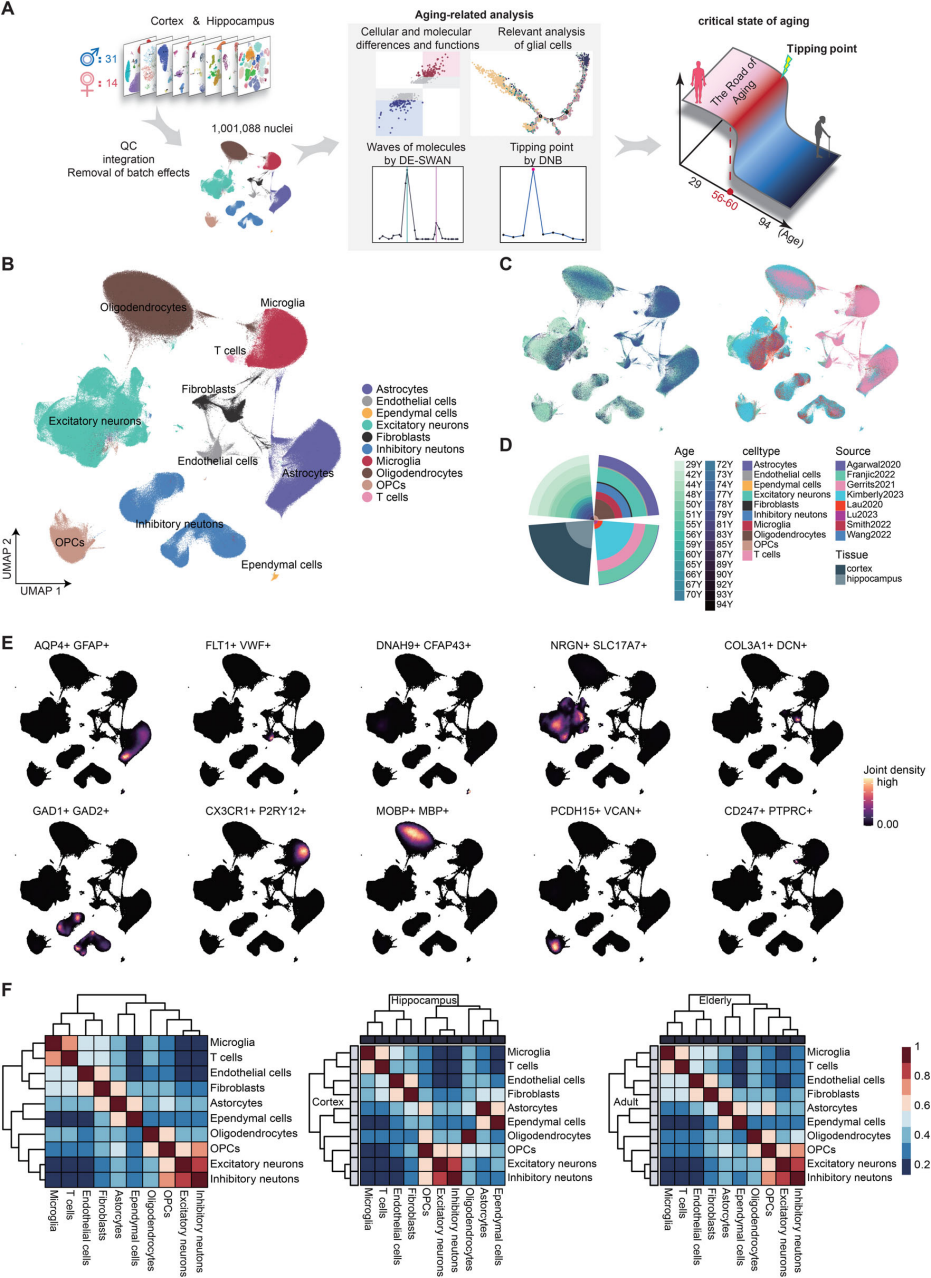
Single‐cell atlas of the healthy aging human brain. A) Detailed description of the data integration, sample information, and research design of this study. B,C) UMAP visualization of all 1 001 088 nuclei in cell type (astrocytes *n* = 172 571; endothelial cells *n* = 15 972; ependymal cells *n* = 2185; excitatory neurons *n* = 219 154; fibroblasts *n* = 22 444; inhibitory neutons *n* = 130 127; microglia *n* = 115 559; oligodendrocytes *n* = 249 254; OPCs *n* = 71 106; T cells *n* = 2716) (B), age samples and data source (C). D) Proportional distribution of cell numbers in different metadata annotation groups. E) Joint density of representative markers for each cell type. F) Correlations between cell types in different samples were analyzed using Spearman's correlation.

To explore the transcriptomic landscape of the human brain from adulthood to aging, we collected single‐cell nuclear transcriptome sequencing data of the human brain with long age spans and large cell numbers through published datasets (the data were mainly obtained from the Gene Expression Omnibus [GEO] and CELLxGENE databases) (Table S1, Supporting Information). Then we independently analyzed the datasets from different sources, selecting and sorting out the cells based on factors such as phenotypes and organization cells eligible for this study. The final data cover cortical and hippocampal samples from 45 healthy humans, spanning from 29 to 94 years of age, and contain 29 age data points. For subsequent analysis, we initially defined age samples from 29 to 60 years old as the young group (adult) and age samples from 65 to 94 years old as the old group (elderly) according to international standards.^[^ ^16^, ^17^, ^18^ ^]^ Finally, after filtering and quality control, a total of 17 145 genes and 1 001 088 high‐quality cell nuclei were obtained for analysis (**Figure** **1**; Figure S1, Supporting Information).

First, we observed a substantial overlap of differentially expressed genes (DEGs) between the cortex and hippocampus during aging, with 41% of all DEGs, 35% of upregulated DEGs, and 39% of downregulated DEGs shared across the two regions (Figure S1C, Supporting Information). This high degree of concordance suggests that aging drives broadly similar transcriptional changes in both brain regions, which supports the rationale for integrating cortical and hippocampal data in our subsequent analysis. Considering the complexity of the sample sources, we then used Harmony for batch processing, and the results showed no specific aggregation between cells from different sources, platforms, and tissues.^[^ ^19^ ^]^ At the level of a cell type annotation, we combined various methods such as singleR software annotation, the CellMarker 2.0 database, and manual annotation of established classical markers from previous research.^[^ ^20^, ^21^, ^22^, ^23^ ^]^ Finally, we identified excitatory neurons (marker: SLC17A7), inhibitory neurons (marker: GAD1), microglia (marker: CX3CR1) (marker: GFAP), astrocytes (marker: GFAP), oligodendrocytes (marker: MBP), oligodendrocyte precursor cells (OPCs) (marker: MEGF11), endothelial cells (marker: VWF), fibroblasts (marker: DCN), ventricular membrane cells (marker: CFAP43), and T cells (marker: CD247) totaling 10 major cell types (Figure 1B,E). Among them, three kinds of glia accounted for ≈53.7% and neurons accounted for ≈34.9% (Figure 1D). To compare cell type similarities across brain regions and age groups, multiple correlation calculations were performed using Pearson correlations. The results found overall similarity in the pattern of cell type correlations, suggesting a high degree of conservatism in the function and properties of cell types across brain regions and aging, and speculated that changes in cell numbers associated with aging may be a more significant feature (Figure 1E).

### 2.2 Cellular and Molecular Signatures of the Human Brain before and after Healthy Aging

**Figure 2:**
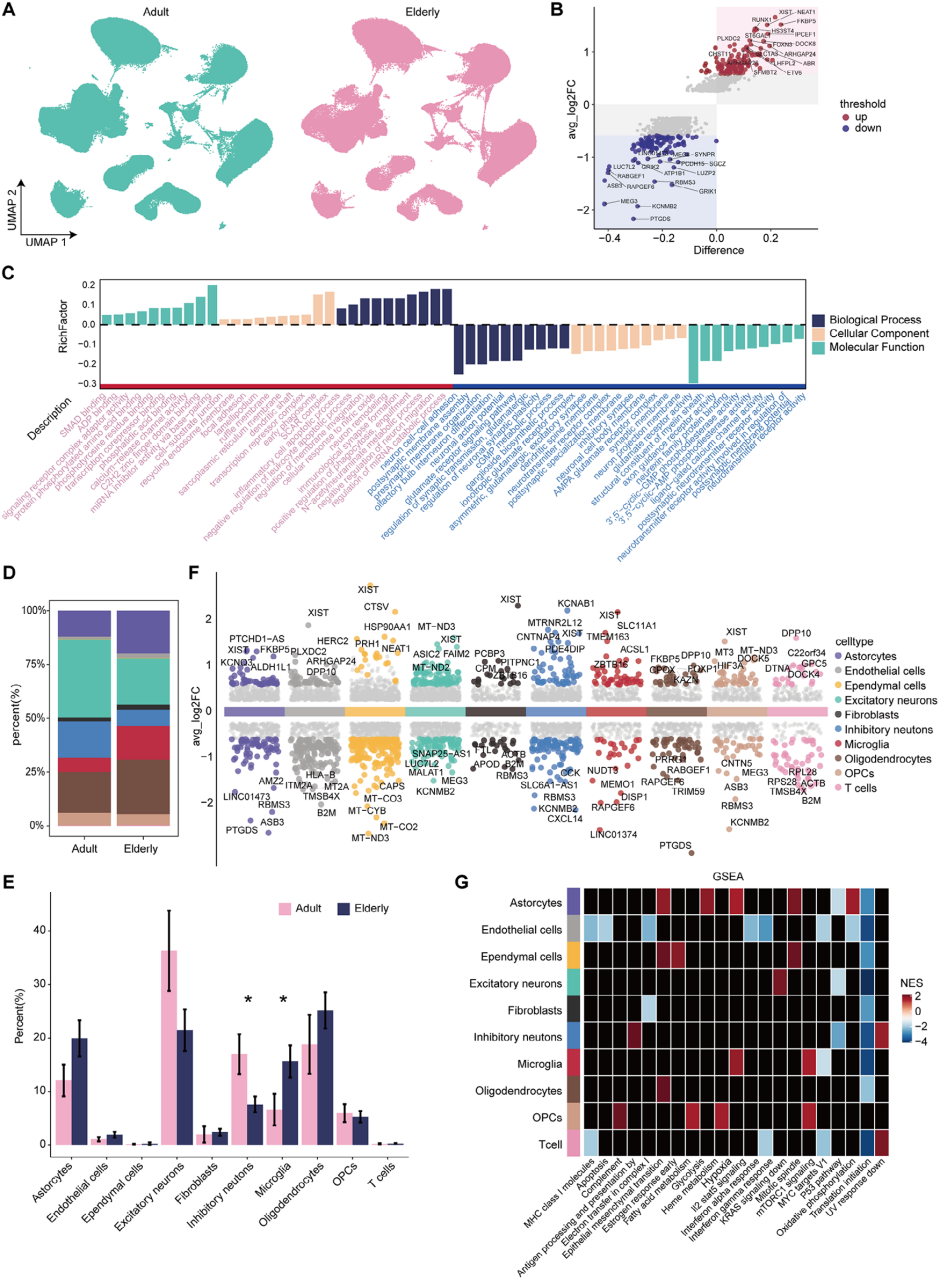
Differences at cellular and molecular level in adult versus elderly groups. A) Differences between adult and elderly groups in UMAP visualization. B) Scatter plot of differential genes. *y*‐axis is log2FoldChange, *x*‐axis is the difference in the proportion of genes expressed in the two groups of cells (Padj < 0.05, FoldChange > 1.5). C) GO enrichment results for up‐ and downregulated genes in elderly. Pink and blue colors correspond to up‐ and downregulated entries (*P*adj < 0.05). D,E) Differences in the proportions of each cell type in adult and elderly groups. The proportions are derived from the number (D) and significance statistics (E) (Wilcoxon rank‐sum test, ^*^ *P* < 0.05). F) Differential gene counts in each cell type, with the top five genes labeled. G) Gene set enrichment analysis (GSEA) results demonstrated significant pathways in each cell type. NES > 0 indicates a positive correlation with age and vice versa for a negative correlation (*P* < 0.05).

Next, we went to the overall level to show the differences that emerged at the cellular and molecular level between the aged and young groups. First, we analyzed the difference between adult and elderly single‐cell transcriptomes (**Figure** **2**A) and identified 497 upregulated differential genes and 508 downregulated differential genes in the elderly group (Figure 2B). Among the upregulated gene clusters, XIST, an X chromosome inactivation‐associated gene, has been reported to be significantly upregulated in the aging hypothalamus and hippocampus and is a potential marker of neuronal senescence in females;^[^ ^24^ ^]^ FKBP5 is involved in stress response, inflammation regulation, and the process of neurodegenerative diseases,^[^ ^25^, ^26^ ^]^ and may play a role in regulating aging‐associated secretory phenotypes (SASPs);^[^ ^27^ ^]^ and DOCK8 regulates immune cell migration and function, possibly by influencing immune response and inflammatory response;^[^ ^28^ ^]^ among the downregulated genes there was also downregulation of GRIK1, which is involved in neurotransmission,^[^ ^29^ ^]^ SYNPR, which is related to the function of neural synapses,^[^ ^30^ ^]^ and RBMS3, which is related to RNA binding and transcriptional regulation,^[^ ^31^ ^]^ suggesting the decline of the nervous system in aging. Similarly, Gene Ontology (GO) enrichment revealed that entries for negative regulation of neuronal migration, transcriptional repression, phagocytosis, immune reaction, and inflammatory cell apoptosis were enriched in aging. Functions such as neuronal differentiation, synapse assembly, and synaptic plasticity were affected in aging (Figure 2C).

Different cell types have different functional responsibilities in the aging process, so different cell types were compared between adult and elderly groups. First, the percentage of cell number before and after aging was analyzed, and it was found that the number of neuronal cells decreased and the number of the three types glial cells (astrocytes, microglia, and oligodendrocytes) increased in the elderly group, with microglial cells being the most significant (Figure 2D,F). We also compared the differences between the adult and elderly groups for each of the different cell types, mapping the up‐ and downregulated genes in each cell type (Figure 2E). Interestingly, XIST was a TOP3 upregulated gene for aging in almost all cell types (Figure 2E; Figure S2A and Table S2, Supporting Information). Given that XIST is a sex‐biased gene with higher expression in females (Figure S2B, Supporting Information), we further analyzed its expression specifically in female samples to eliminate potential sex‐related confounding. XIST remained one of the most significantly upregulated genes during aging across multiple cell types, including excitatory neurons, inhibitory neurons, and endothelial cells (Figure S2C,D, Supporting Information), further supporting its role as a female brain and cell type‐specific aging marker, consistent with previous findings.^[^ ^24^ ^]^ Further using Gene Set Enrichment Analysis (GSEA) on the differential genes of each cell type, we obtained relatively independent senescence‐related features for different cell types (Figure 2G). For example, the enrichment of the mTORC1 pathway in microglia and OPCs, where previous studies have found that mTORC1 overactivation inhibits autophagy, leading to intracellular accumulation of damage.^[^ ^32^ ^]^ The enrichment of the IL2‐STAT5 signaling pathway in microglia and astrocytes also suggests the influence of both on the regulation of immune cell proliferation and differentiation in the senescent state.^[^ ^33^ ^]^ In addition, it is noteworthy that almost all senescent cell types exhibited downregulation of translation initiation, suggesting that impaired translation initiation leading to decreased efficiency of protein synthesis is a universal feature of the senescence process.

### 2.3 Revealing Nonlinear Molecular Waves during the Aging Process at Single‐Cell Resolution

To understand the quantitative changes in molecules over the life cycle with age, we calculated the linear correlations between their expression levels and age using linear regression for all molecules across every cell. Our analysis revealed that most genes exhibit nonlinear expression changes with age, except for a few genes that show weak correlations (Figure S2E and Table S3, Supporting Information).

To pinpoint the specific periods of dysregulation for these molecules during aging, we employed a differential expression sliding window analysis (DE‐SWAN),^[^ ^11^ ^]^ which employs a sliding window of 20 years, with multiple ages ranging from 29 to 94 as the center of the window, and progressively along the direction of age increase for sliding comparisons. When we used *p*‐values to define significance, peaks were identified to occur at ages 60, 70, and 79, and when adjusted *p*‐values were used, peaks similarly occurred at ages 60 and 79 (Figure S3A, Supporting Information). We also detected wave crests in different cell types that exhibited patterns consistent with the overall trends (Figure S3C, Supporting Information), indicating the stability and reliability of these aging‐related waves. Furthermore, the representative molecules associated with these waves highlight distinct molecular characteristics corresponding to different aging stages (Figure S3B, Supporting Information).

### 2.4 Glia Exhibit a Higher Correlation with the Aging Process

**Figure 3:**
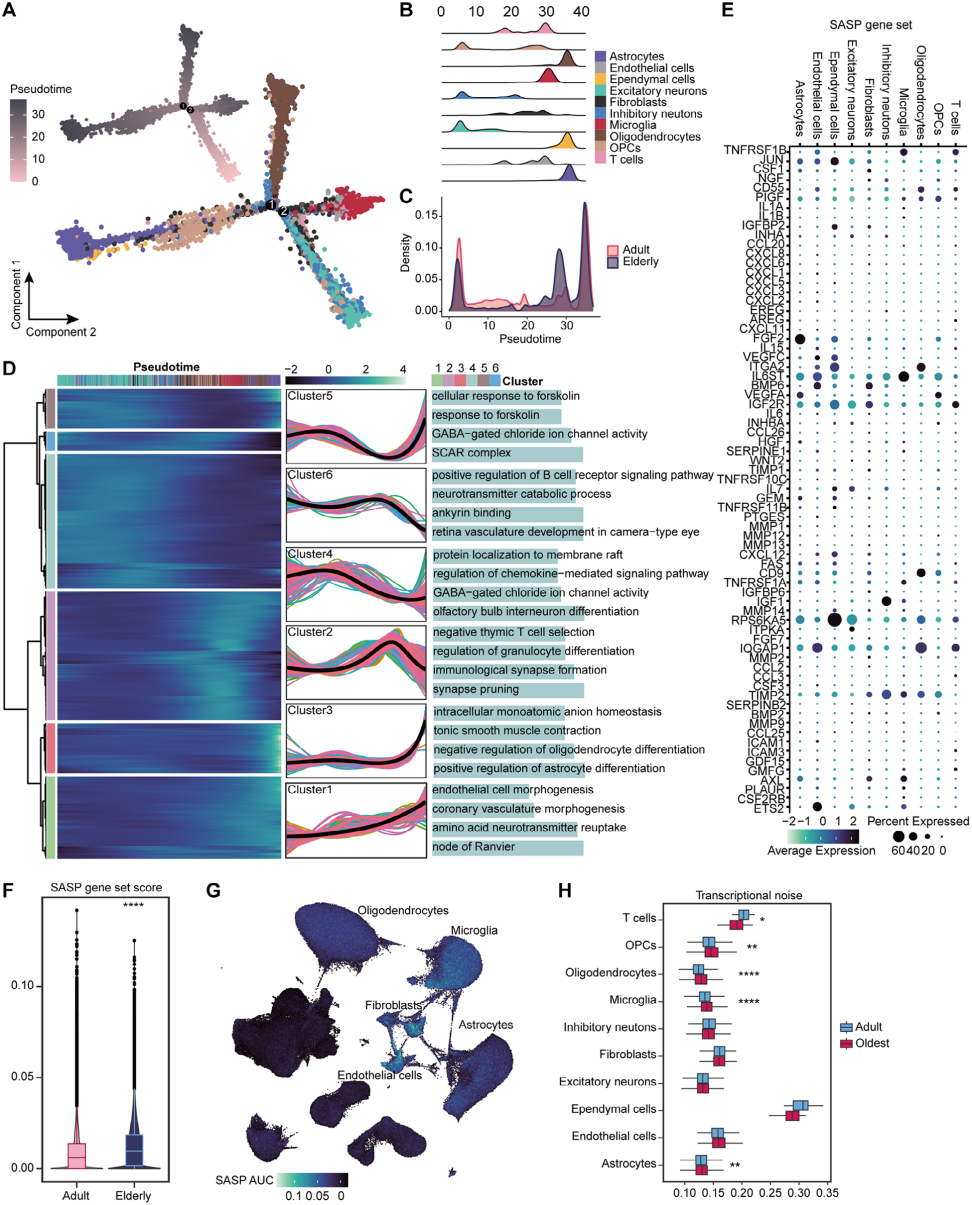
Construction of cell type developmental trajectories and assessment of senescence correlations. A) Pseudotemporal ordering of all cell types. B,C) Distribution of cell types (B) and age groups (C) on the pseudotime. D) Clustering and functional annotation of all genes based on their expression changes on pseudotime. E) Expression levels of members of the senescence‐associated secretory phenotype (SASP) in different cell types. F,G) SASP gene set scores by age group (F) and cell type (G). H) Box plots showing transcriptional noise levels for each cell type in the adult (29–60 years) and oldest (77–90 years) groups (Wilcoxon rank‐sum test, ^*^ *P* < 0.05,^**^ *P* < 0.01,^***^ *P* < 0.001,^****^ *P* < 0.0001).

Glial cells not only support and nourish neurons but also play a key role in essential brain functions, including signaling, neuroprotection, immune response, and synaptic regulation.^[^ ^21^, ^34^, ^35^ ^]^ Increasing research highlights that dysfunction and aberrant activation of glia with age significantly contribute to neurological disorders and aging.^[^ ^36^, ^37^, ^38^ ^]^ Given the prominent quantitative changes and associated functional alterations in glia during aging (Figure 2D–G), we further analyzed them using the pseudotime approach. The distribution of cell types on the trajectories was consistent with previous reports,^[^ ^22^ ^]^ with excitatory neurons, inhibitory neurons, and OPCs appearing at the onset of development, beginning to form in early embryonic and fetal stages of development, and progressively diverging into three branches toward microglia, astrocytes, and oligodendrocytes as development advanced (**Figure** **3**A,B). Due to the diversity and complexity of cell states in individuals of different ages, the adult and elderly groups did not exhibit clear polarization in the distribution of the pseudotime trajectories (Figure S4A, Supporting Information), Nevertheless, there was still a general trend of a gradual decreased cell numbers in the adult group and an increased cell numbers in the elderly group along the trajectories (Figure 3C). We then clustered the gene expression along the developmental trajectory into six clusters, and using GO enrichment, found that functional changes with increasing pseudotime closely mirrored age‐associated functional characteristics (Figure 3D). These results suggest that alterations in synaptogenesis, neurodevelopmental regulation, and immune and inflammatory responses with age are closely linked to changes in glia function.

The senescence‐associated secretory phenotype (SASP), characterized by the secretion of proinflammatory cytokines, chemokines, growth regulators, angiogenic factors, and matrix metalloproteinases, is a hallmark of senescent cells.^[^ ^39^ ^]^ We utilized SASP to assess senescence both overall and by cell type (Table S4, Supporting Information). The scores were calculated using the AUCell algorithm to reflect the relative transcriptional activity of SASP‐associated genes in each cell. Our analysis revealed that the SASP score was significantly elevated in the elderly group. UMAP analysis further demonstrated that three types of glia, fibroblasts, and endothelial cells exhibited higher and more intense SASP scores (Figure 3F,G). The expression of different functional SASP genes within each cell type is also shown (Figure 3E). Similar trends were observed when scoring the gene set associated with the classical senescence phenotype (CSP)^[^ ^40^ ^]^ (Figure S4B–D and Table S4, Supporting Information). The CSP gene set includes genes involved in canonical senescence pathways, such as CDKN1A, TP53, and components of the DNA damage response.

As cells age, their transcriptional regulation becomes increasingly unstable, and the accumulation of “junk” mRNAs inevitably impacts cellular function.^[^ ^41^ ^]^ We observed a rise in the coefficient of variation with age across nearly all cell types, indicating variability in gene expression during aging (Figure S4E, Supporting Information). Moreover, we applied a transcriptional noise calculation method to quantify transcriptional instability across different cell types.^[^ ^42^ ^]^ By analyzing several age groups, we found that transcriptional noise increased more markedly with age in microglia, astrocytes, and oligodendrocytes, consistent with previously reported findings^[^ ^43^ ^]^ (Figure S4F,G, Supporting Information). In contrast, neurons exhibit weaker senescence correlation and stability during aging.

Overall, we present a comprehensive cellular and molecular landscape of brain aging, in which the three types of glia exhibit a stronger correlation with aging, encompassing aspects such as cell number, molecular expression, functional enrichment, and the assessment of both development and aging. Further, we will investigate the aging landscape of these three glia in greater detail.

### 2.5 Microglia Tipping Point Age Prediction during Aging and Senescence Biomarkers Identification

**Figure 4:**
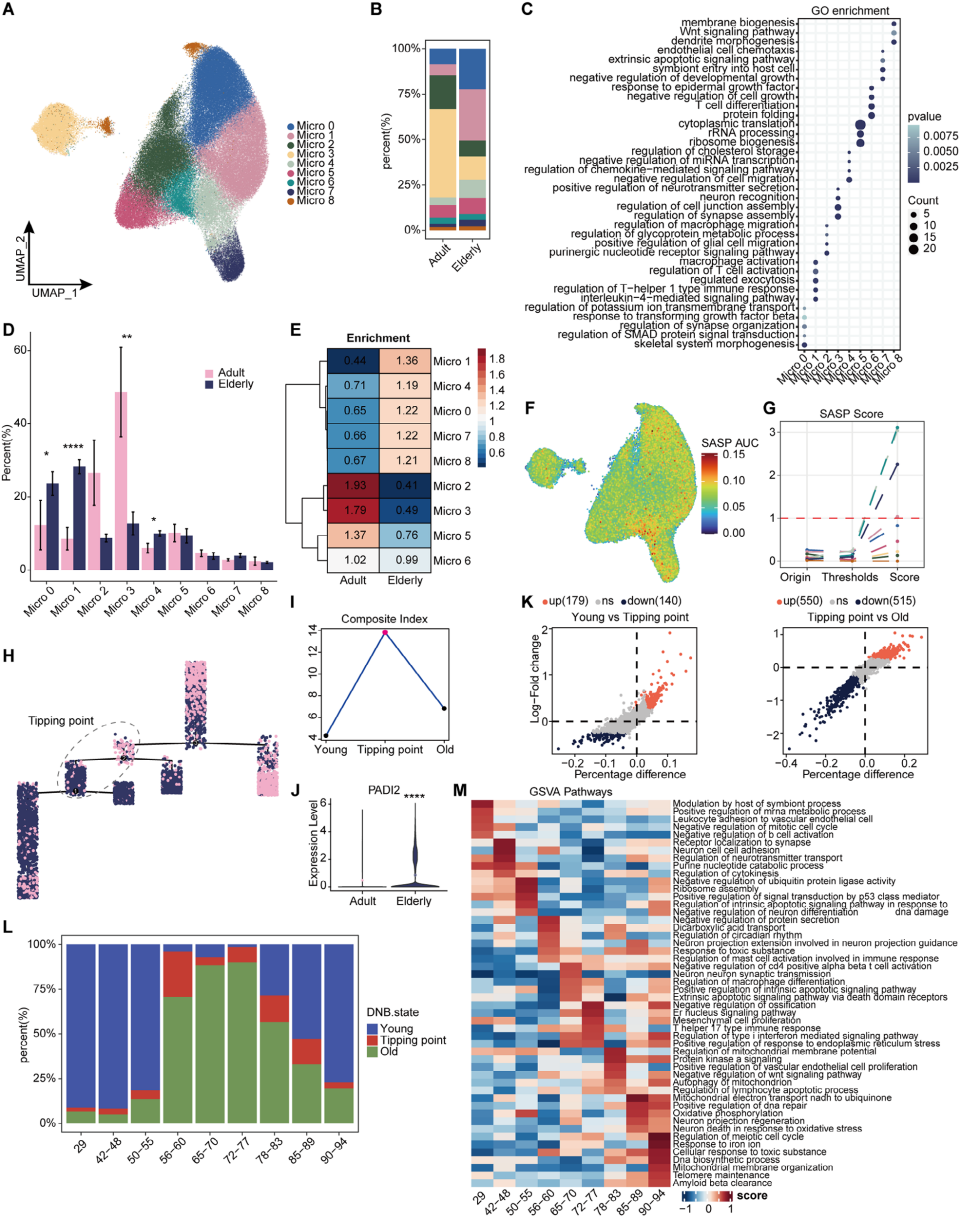
Subtype differences and nonlinear dynamics characteristics of microglia during senescence. A) Microglia subtypes identified (*n* = 115 559). B) Proportion of cell counts in age groups for each subtype. C) GO enrichment analysis demonstrates functional differences between microglia subtypes. D) Statistical results of the significance of the number of cells of each subtype as a percentage of age groups (Wilcoxon rank‐sum test) ^*^ *P* < 0.05,^***^ *P* < 0.001,^****^ *P* < 0.0001). E) Sample enrichment analyses identified enrichment scores for each subgroup in age phenotypes. F,G) The SASP gene set for microglia subtypes was scored (F) and the new SASP score was quantified by the change in the proportion of cells from initial to threshold reached (G). H,I) Constructing pseudotime trajectories by microglia subtypes and coloring cells according to age groups (H)) and combined with the DNB calculation method) the tipping point of the senescence critical state was found and the cells before and after being in this state on the pseudotime were defined as young and old state (I). J) PADI2, a key gene in dynamic molecular networks) was significantly upregulated in expression in elderly group (^****^ *P* < 0.0001). K) Differences between the three states of cells as defined by DNB (log2FoldChange > 0.25). L) Distribution of cells in the three DNB states at different ages. M) The highest scoring terms for each age group were selected as the primary functional traits using gene set variance analysis (GSVA).

Microglia exhibited the highest correlation with senescence in the previous result. To further explore this, we subdivided microglia into nine distinct subtypes (**Figure** **4**A), markers of each subtype are shown in the figure (Figure S5A, Supporting Information). Several subtypes demonstrated significant differences in cell number before and after aging, such as Micro0, 1, and 4, which showed a notable increase in the proportion of cells in the elderly group, while Micro3 showed a significant decrease with aging (Figure 4B,D). Additionally, the functional characteristics of these subtypes revealed significant differences (Figure 4C). We also examined the correlations between subtypes across overall, adult, and elderly samples (Figure S5B, Supporting Information). Notably, in the elderly group, Micro1, 0, 4, 6, and 2 displayed distinct changes compared to the overall and adult groups, with a high degree of correlation observed between these subtypes.

To further identify the subtype most closely associated with aging, we performed sample enrichment analysis for each subtype (Figure 4D,E). Our analysis revealed that Micro3 was more closely associated with younger samples, while Micro0, 1, and 4 were more abundant in aging tissues. Using SASP and senescence markers, we assessed the senescence status of each subtype (Figure 4F,G; Figure S5E, Supporting Information). Micro4, 6, and 7 exhibited extremely high senescence scores, with Micro1 following closely behind. Moreover, comparing the differences between adult and elderly groups (Figure S5C,D, Supporting Information) revealed significant overlap in the differential gene functions of Micro1, 4, 6, and 7. In summary, we propose that these four microglia subtypes are most strongly associated with aging. Their involvement in the aging process is primarily through regulating immune responses, cellular homeostasis, apoptosis, and other functions (Figure 4C). It is also worth noting that an increasing body of research has highlighted the link between microglia and neurodegenerative diseases, which contribute to memory, learning, and cognitive decline.^[^ ^38^ ^]^ Using a set of learning and memory‐related genes for scoring (Table S4, Supporting Information), we found that Micro3, significantly downregulated in senescent tissues, was the only subtype to achieve exceptionally high scores. This finding aligns with GO analysis, suggesting that Micro3 maintains learning and memory by regulating processes such as synapse assembly and neuron recognition, and is more strongly affected in senescent tissues (Figure 4C).

The unsupervised fuzzy c‐means clustering approach also confirmed the nonlinear nature of age‐dependent changes in microglia (Figure S5I, Supporting Information). Studies have increasingly highlighted the “cliff‐like” aging characteristics observed at certain ages, which have driven us to consider brain aging as a nonlinear process driven by critical states. We explored this using kinetic systems theory and methodology. We applied the theory of DNBs to predict abrupt, irreversible transitions in aging states at critical points in a dynamic process, driven by sudden shifts in the molecular state at the core of the system.^[^ ^15^ ^]^ First, we constructed the developmental trajectories of the Micro1, 3, 4, 6, and 7 subtypes, which are strongly associated with senescence, to remove noise caused by different cell types (Figure 4H; Figure S5G, Supporting Information). We then introduced the DNB to calculate the composite index (CI), combining developmental bifurcation and the phenotypic distribution of young and aged samples, successfully predicted the peak of the critical state in the young‐to‐aging process (Figure 4I; Figure S5H, Supporting Information), the potential tipping point of aging. The tipping point cells were found to occur primarily between the ages of 56–60, where the number of young cells sharply declined while senescent cells increased significantly (Figure 4L). Through gene set variation analysis at each stage, we observed that adaptive regulations such as homeostatic maintenance and damage repair were more prevalent before the age of 56–60. However, at 56–60, blocked protein secretion, circadian rhythm dysregulation, and immune response to toxic substances led to an irreversible transition toward functional imbalance.^[^ ^44^ ^]^ After the age of 56–60, the cumulative effects ofaging became more apparent, with degeneration, apoptosis, and tissue repair coexisting (Figure 4M). Further analysis of the three DNB states and functional analysis of the tipping point revealed a similar tipping point at ages 56–60 (Figure 4K; Figure S6A and Table S5A,B, Supporting Information). Transcriptional noise analysis also revealed peaks of transcriptional instability at ages 56–60 and 78–83 (Figure S6B, Supporting Information), which aligns with the peak distribution of molecular waves (Figure S3C, Supporting Information).

Additionally, we mapped the molecular networks critical for the tipping point, with mTOR showing the most significant and important connections (Figure S5J, Supporting Information). It has been shown that inhibiting mTOR can extend the lifespan of model organisms.^[^ ^45^, ^46^, ^47^ ^]^ Moreover, we identified the PADI2 gene (Figure 4J), one of the Top 10 genes upregulated in microglia during aging, consistent with previous findings in primates.^[^ ^43^ ^]^ hdWGCNA analysis revealed that PADI2 is part of module 4 (Table S7A, Supporting Information), which is highly correlated with the aging phenotype. Notably, in time series analysis, PADI2 clustered with XIST, TP53, and other aging markers in cluster 3 (Table S6A, Supporting Information), and the gene expression of this cluster exhibited a sharp increase after the age of 56–60, suggesting that PADI2 may serve as a potential aging marker (Figure 4J; Figure S5I–L, Supporting Information).

In summary, we found and demonstrated the microglia subtypes and its nonlinear dynamic characteristics in aging, and concluded that 56–60 years old is an important critical inflection point age stage for microglia‐driven dramatic aging.

### 2.6 Astrocyte Tipping Point Age Prediction during Aging and Senescence Biomarkers Identification

**Figure 5:**
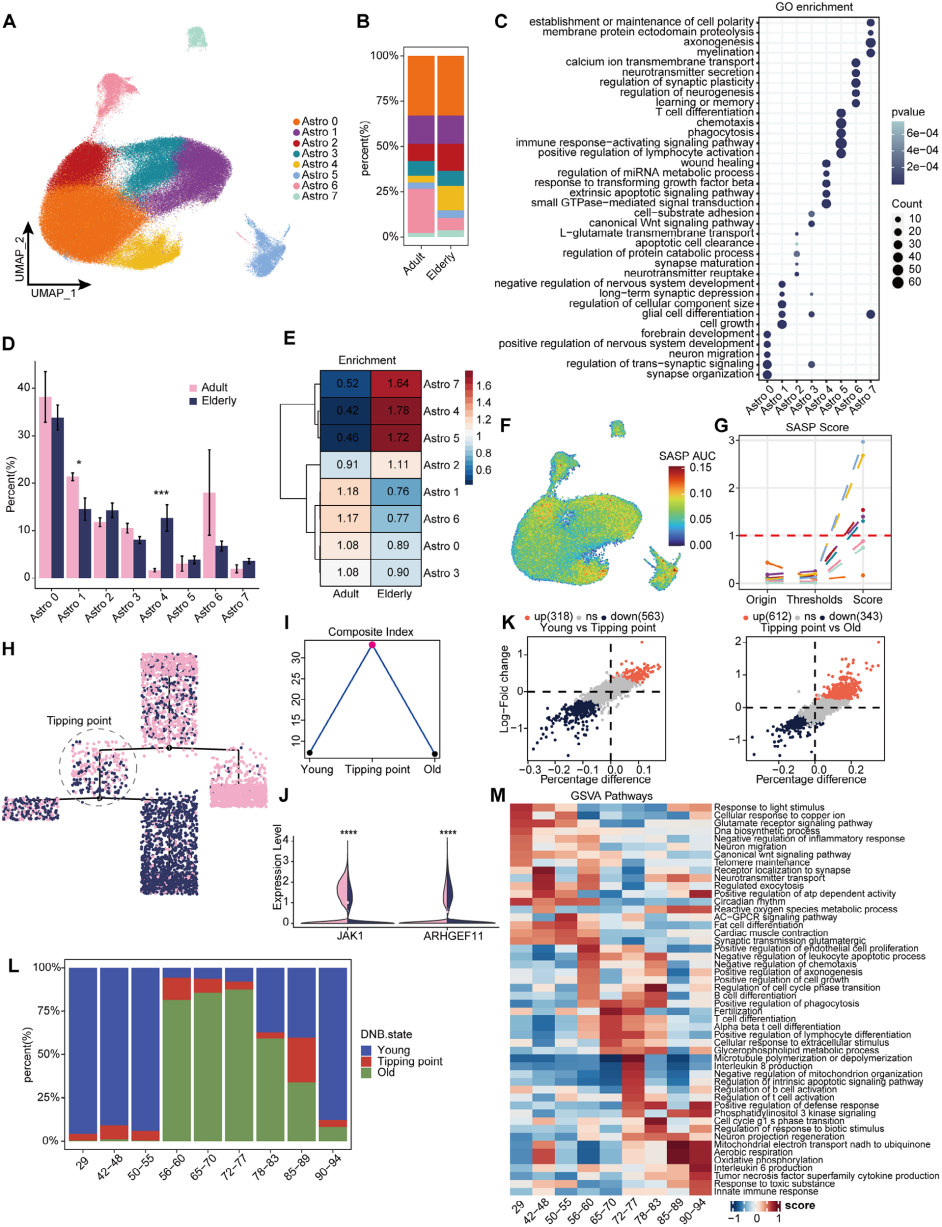
Subtype differences and nonlinear dynamics characteristics of astrocytes during senescence. A) Astrocyte subtypes identified (*n* = 172 571). B) Proportion of cell counts in age groups for each subtype. C) GO enrichment analysis demonstrates functional differences between astrocyte subtypes. D) Statistical results of the significance of the number of cells of each subtype as a percentage of age groups (Wilcoxon rank‐sum test, ^*^ *P* < 0.05, ^***^ *P* < 0.001). E) Sample enrichment analyses identified enrichment scores for each subgroup in age phenotypes. F,G) The SASP gene set for astrocyte subtypes was scored (F) and the new SASP score was quantified by the change in the proportion of cells from initial to threshold reached (G). H,I) Constructing pseudotime trajectories by astrocyte subtypes and coloring cells according to age groups (H), and combined with the DNB calculation method, the tipping point of the senescence critical state was found and the cells before and after being in this state on the pseudotime were defined as young and old state (I). J) JAK1 and ARHGEF11, key genes in the DNB network, were significantly downregulated and upregulated in elderly group, respectively (^****^ *P* < 0.0001). K) Differences between the three states of cells as defined by DNB (log2FoldChange > 0.25). L) Distribution of cells in the three DNB states at different ages. M) The highest scoring terms for each age group were selected as the primary functional traits using gene set variance analysis (GSVA).

We further identified eight different astrocyte subtypes (**Figure** **5**A; Figure S7A, Supporting Information). Upon analyzing the changes in their percentage, we found that the ratios of Astro1 and Astro6 were downregulated in senescent tissues. Astro1 was mainly associated with cell growth and glial cell differentiation, while Astro6 was primarily linked to learning, memory, and the regulation of synaptic and neurotransmitter secretion. Notably, scoring of the learning and memory gene sets indicated that Astro6 is the only astrocyte isoform strongly associated with learning and cognitive functions (Figure S7F, Supporting Information). Astro4 was significantly upregulated in senescent tissues, with functional enrichment in apoptosis, the TGF‐β pathway, and wound healing (Figure 5B–D). A marked difference in its abundance before and after aging was observed (Figure S7B, Supporting Information). Additionally, Astro5 exhibited a high enrichment score for the senescence phenotype, with functions closely related to immunity and phagocytosis (Figure 5C,E). The SASP score and the distribution of CDKN2A/CDKN1A further confirmed the strong correlation between Astro1, 5, and 4 with senescence (Figure 5F,G; Figure S7E, Supporting Information). The nonlinear molecular features were also captured in astrocytes (Figure S7I, Supporting Information). We then constructed pseudotime trajectories for Astro1, 4, 5, and 6, with Astro6 at the beginning and Astro5 at the end of the developmental trajectory (Figure S7B, Supporting Information). The distribution patterns of the younger and older groups also displayed a clear difference over time (Figure 5H).

We performed DNB calculations on each state cell cluster along the trajectory and found that one of the overbranching points between the adult and elderly samples exhibited the highest critical score (Figure 5I; Figure S7H, Supporting Information), corresponding to the tipping point of astrocytes. Upon analyzing the molecular and functional differences between the three DNB states (Table S5C,D, Supporting Information), we observed that, compared to the young state, the tipping point primarily exhibited upregulation of responses to stimuli and injury, along with downregulation of functions such as oxidative phosphorylation, ribosomal genesis, and synaptic regulation. Once the aging state was reached, immune and phagocytic response modulation was enhanced, while brain development, neurogenesis, and synaptic regulation were further impaired (Figure 5K; Figure S6C, Supporting Information). Tipping point cells were predominantly enriched at ages 56–60 and 85–89, similar to microglia, there is a marked increase in senescent state cells observed at 56–60 years, likely due to the overactivation of immune‐inflammatory response (Figure 5L,M).

The DNB molecular network identified JAK1 and ARHGEF11 as potential key genes in the tipping point (Figure 5J; Figure S7I–L, Supporting Information). JAK1 was found to be downregulated during senescence, with Mfuzz analysis placing it in cluster 5, where its expression sharply declines between the ages of 56 and 60. Additionally, hdWGCNA analysis revealed that JAK1 is located in Module 4 (Table S7B, Supporting Information), which shows a negative correlation with age. Inhibiting JAK1 has been shown to reduce SASP secretion in senescent cells, effectively delaying aging.^[^ ^48^, ^49^ ^]^ In contrast, ARHGEF11 acts as a guanine exchange factor for RhoA, regulating cell proliferation, migration, and epithelial–mesenchymal transition.^[^ ^50^, ^51^ ^]^ It was upregulated during aging, classified in cluster 9, and exhibited a sustained increase after the age of 56–60. It is part of Module 11 (Table S7B, Supporting Information), which is positively correlated with age. Notably, CDKN1A, CDKN2A, and PADI2 were also identified in cluster 9 (Table S6B, Supporting Information), further underscoring the potential importance of these genes in the aging process.

In summary, our study uncovered the cellular and molecular heterogeneity of astrocytes during aging, highlighting their nonlinear dynamics throughout the aging process. We also identified key cell clusters in tipping point. Similar to microglia, we propose that the age range of 56–60 years represents a pivotal period for astrocytes, during which systemic dysregulation occurs, driving a significant shift in cell states toward senescence.

### 2.7 Oligodendrocyte Tipping Point Age Prediction during Aging and Senescence Biomarkers Identification

**Figure 6:**
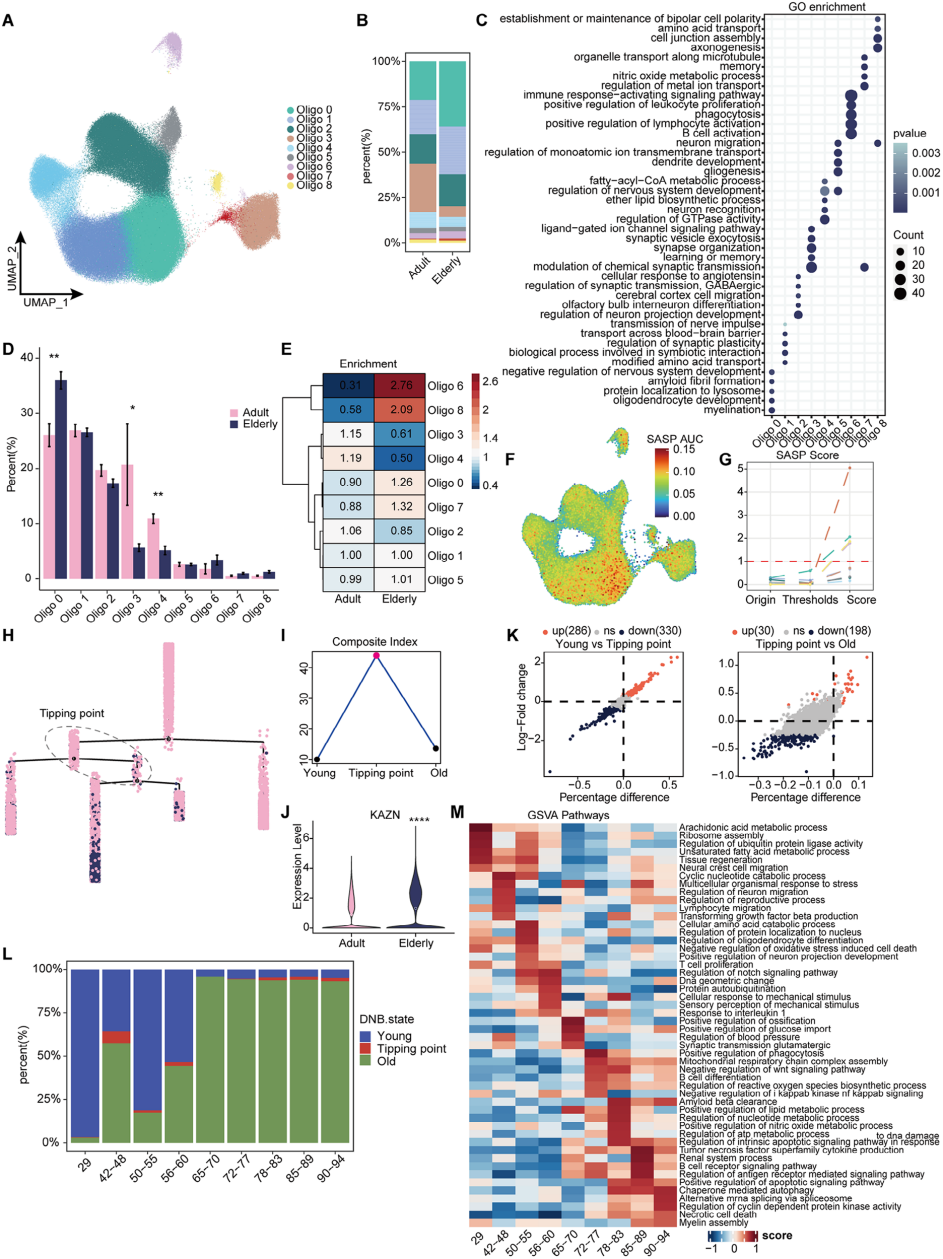
Subtype differences and nonlinear dynamics characteristics of oligodendrocytes during senescence. A) Oligodendrocytes subtypes identified (*n* = 249 254). B) Proportion of cell counts in age groups for each subtype. C) GO enrichment analysis demonstrates functional differences between oligodendrocytes subtypes. D) Statistical results of the significance of the number of cells of each subtype as a percentage of age groups (Wilcoxon rank‐sum test, ^*^ *P* < 0.05, ^**^ *P* < 0.01). E) Sample enrichment analyses identified enrichment scores for each subgroup in age phenotypes. F,G) The SASP gene set for oligodendrocytes subtypes was scored (F) and the new SASP score was quantified by the change in the proportion of cells from initial to threshold reached (G). H,I) Constructing pseudotime trajectories by oligodendrocytes subtypes and coloring cells according to age groups (H), and combined with the DNB calculation method, the tipping point of the senescence critical state was found and the cells before and after being in this state on the pseudotime were defined as young and old state (I). J) KAZN, a key gene in the DNB network, were significantly upregulated in elderly group (^****^ *P* < 0.0001). K) Differences between the three states of cells as defined by DNB (log2FoldChange > 0.25). L) Distribution of cells in the three DNB states at different ages. M) The highest scoring terms for each age group were selected as the primary functional traits using gene set variance analysis (GSVA).

We identified nine oligodendrocyte subtypes (**Figure** **6**A; Figure S8A, Supporting Information). Among these, Oligo0 was significantly upregulated in aging tissues, primarily linked to myelination and oligodendrocyte development. In contrast, Oligo3 and Oligo4 were markedly downregulated with aging, with Oligo3 associated with synaptic modulation and memory, and Oligo4 with neuronal recognition and nervous system development. Oligo6 showed a pronounced increase in aging, enriched for immune‐related functions such as B‐cell activation and leukocyte proliferation (Figure 6B–D). Its lower similarity to other subtypes suggests functional specificity (Figure S8B, Supporting Information). In Difference analysis, Oligo6 exhibited the greatest differences pre‐ and postaging (Figure S8C,D, Supporting Information). Enrichment analysis revealed that Oligo0, 6, and 8 were more associated with senescence, while Oligo3 and Oligo4 aligned with youthful phenotypes. Senescence scoring identified Oligo0, 3, 4, and 6 as the most age‐associated subtypes (Figure 6F,G; Figure S8E,F, Supporting Information).

Given that oligodendrocytes also exhibit molecular nonlinearity, we proceeded to use a combination of developmental trajectory construction and DNB computation and found tipping point cell clusters (Figure 6H,I; Figure S8G–I, Supporting Information). Differential analysis of the three DNB states revealed that the tipping point cells were less distinct from aged cells (Figure 6K; Table S5E,F, Supporting Information) but functionally more divergent compared to young cells. In the tipping point, oligodendrocytes exhibited impaired ribosomal genesis, cytoplasmic translation, and oxidative phosphorylation, while maintaining neuronal migration, glial differentiation, and synaptic organization. Aged‐state cells showed heightened immune stress and apoptosis, along with disrupted myelination and neural regeneration (Figure S6E, Supporting Information). Age‐dependent analysis revealed oligodendrocyte instability as early as 42–48 years, marked by altered cyclic nucleotide metabolism, neuronal migration, and lymphocyte migration. After 65–70 years, senescent cells accumulated significantly, responding to aging through mitochondrial regulation, immune‐inflammatory responses, and damage repair (Figure 6L,M, Supporting Information).

We also constructed a DNB molecular network and identified KAZN, which was significantly upregulated in the senescent group and appeared in age‐related module 2 of the hdWGCNA results (Figure 6J; Figure S8J–L and Table S7C, Supporting Information). This gene encodes kazrin, a protein involved in desmosome assembly and adherens junctions.^[^ ^52^ ^]^

### 2.8 Glia Mediate the Tipping Point at 56–60 Years in the Healthy Aging Brain

**Figure 7:**
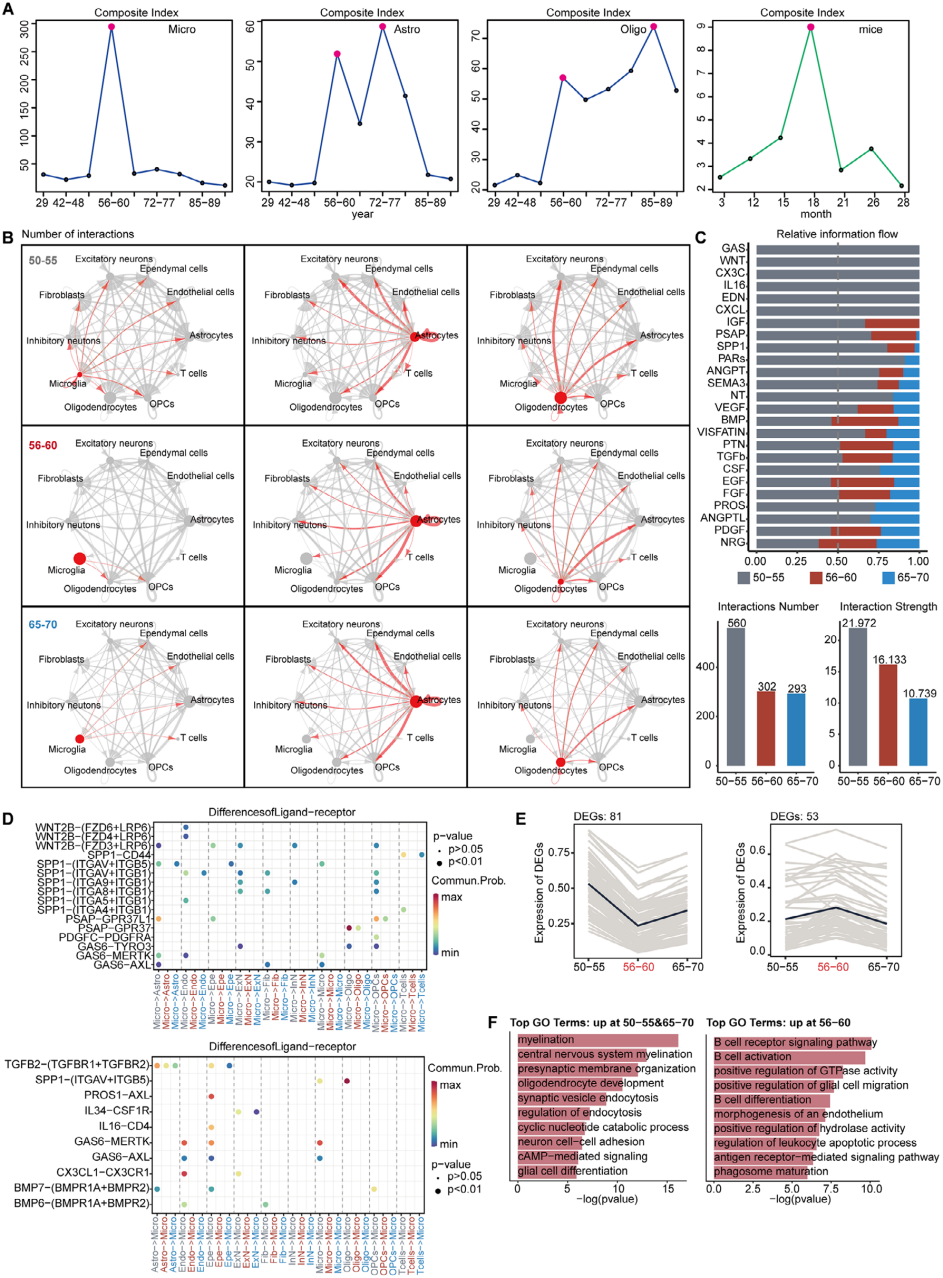
Critical peaks and complex pathway changes in glia at age 56–60 strongly support it as a critical period of brain aging. A) Age‐related tipping point in glia and mouse data. B) Cellular communication of glia at 56–60 years of age was significantly different from that of adjacent age groups 50–55 and 65–70 years of age. C) Identification of conserved and specific signaling pathways at three ages, vertical dashed lines indicate where samples account for 50% of the overall information flow. D) Signaling pathways involved in communication with other cell types when microglia act as ligands and receptors (*P* < 0.01). E) Trends and number statistics of down‐ and upregulated differential gene expression at ages 56–60 compared to the other two age groups. F) Functional annotation of down‐ and upregulated differential genes in the 56–60 age group (*P* < 0.05).

Next, we applied DNB scoring to all cells across all ages without senescence‐related screening and identified specific tipping point peaks in all cell types (**Figure** **7**A; Figure S9A, Supporting Information). More interestingly, the three glial cell types exhibited tipping points earliest, and all appeared at 56–60 years, consistent with the above findings. Integrating DE‐SWAN analysis and prior results, we propose that a tipping point driving accelerated brain aging emerges at 56–60 years, and this is likely driven by functional changes in glia. Transcriptional noise calculations were again used to quantify transcriptional instability at various ages, and it was also found that the age of transcriptional instability for the three glia was very similar to the results for their respective DNB findings. Notably, females showed an earlier onset of instability compared to males (Figure S6B,D,F, Supporting Information).

We also validated this in a publicly available RNA‐seq dataset from 59 mice aged 3 to 28 months,^[^ ^53^ ^]^ we extracted cortex and hippocampal tissue data for analysis (Figure S9B, Supporting Information). Consistent with previous findings, Padi2 showed a significant positive correlation with age (Figure S9C, Supporting Information). DNB scoring across all ages revealed a tipping point at month 18 (approximately 56 years in humans), with key DNB molecules enriched in aging and immune‐inflammation pathways (Figure 7A; Figure S9D, Supporting Information).

**Figure 8:**
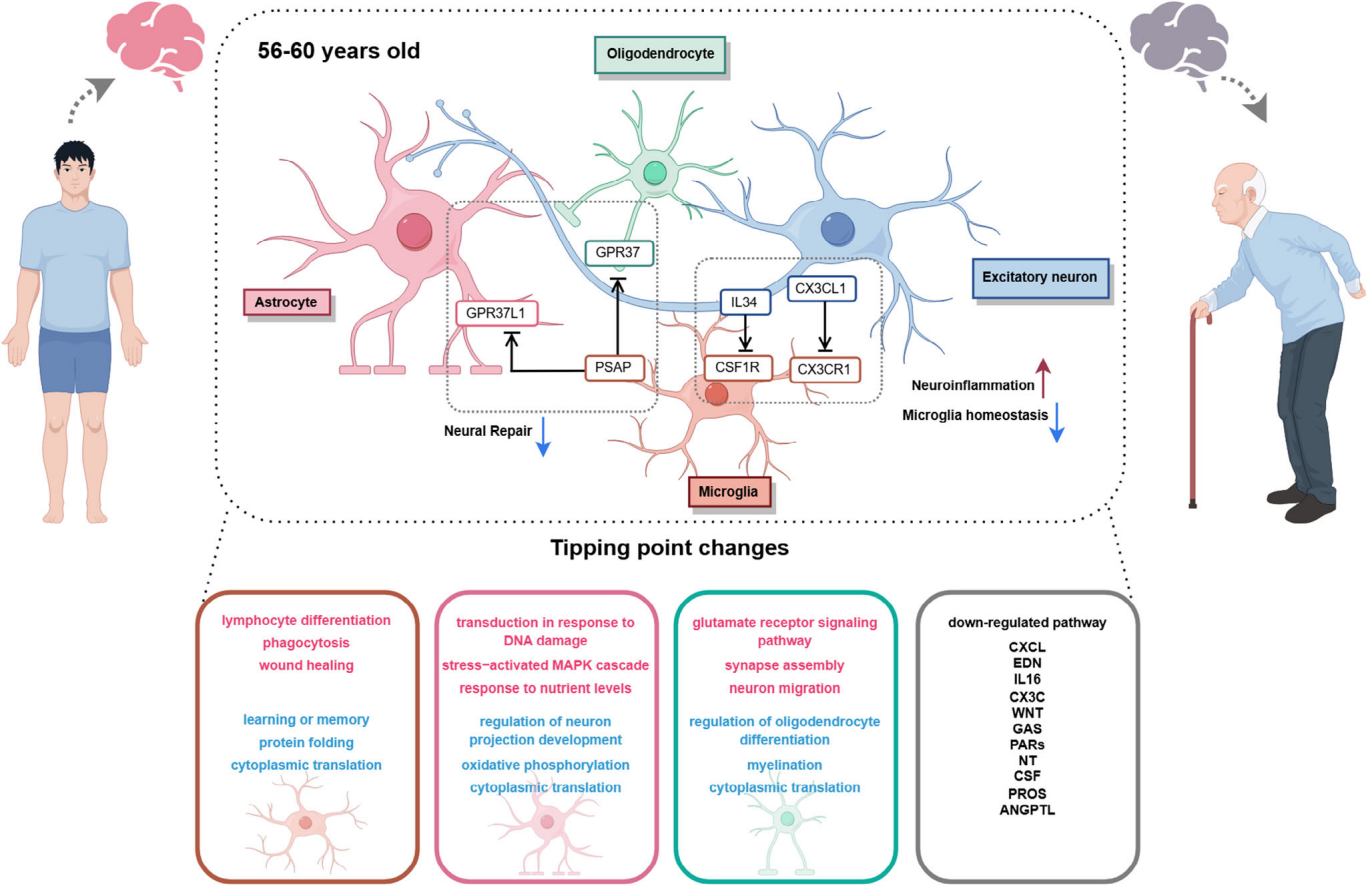
Important cellular functions and communication in the tipping point stage of brain aging. The top part represents the important intercellular communication at the tipping point. Arrow indicates blockage of communication and colored arrows represent functional changes due to ligand–receptor status next to them. The lower part shows representative functional enrichment and important pathways of glial cells at this stage. (Red represents upregulation blue represents downregulation.) This image was drawn by Figdraw.

In order to identify the pathways and functions contributing to the tipping point of irreversible disruption. We selected the 56–60 years group for CellChat analysis. Additionally, ages 50–55 and 65–70 were included for comparison as the pre‐ and post‐tipping point stages. At age 56–60, the three glia types exhibited substantial differences in cellular communication compared to other cell types, primarily manifested by a marked reduction in the number and strength of communication, particularly in microglia (Figure 7B). A total of 25 pathways were involved in intercellular communication across these three age groups, including 13 conserved pathways, 6 specific to age 50–55, 5 shared between 50–55 and 65–70, and 1 shared between 50–55 and 60–65 (Figure 7C). Specifically, when the microglia with the most distinct communication differences act as ligands, cell proliferation, migration, neuroprotection, tissue repair, and angiogenesis, all of which were downregulated at 56–60 years (e.g., GAS, Wnt, PSAP, and SPP1). Notably, weakened communication between three types of glia related to neuroprotective and neural repair functions is more likely to lead to tipping point dysfunction (PSAP‐GPR37L1, PSAP‐GPR37).^[^ ^54^ ^]^ As receptors, microglia also downregulated pathways related to cell proliferation, migration, neural repair, and immunomodulation (e.g., CX3C, CSF, IL16, GAS, and TGFb). Among them, the attenuation of communication between microglia and excitatory neurons via CX3CL1‐CX3CR1 and IL34‐CSF1R may disturb the immune environment during aging, exacerbating tissue damage and apoptosis^[^ ^55^, ^56^, ^57^ ^]^ (Figure 7D). We summarize and present the pathways and functions of key representative cells through schematic diagrams (**Figure** **8**). Furthermore, a differential comparison of the three age stages revealed 81 downregulated genes and 53 upregulated genes at 56–60 years (Figure 7E,F). Key functional alterations included the damage of myelin formation, synaptic organization, and cell adhesion regulation, along with enrichment of B‐cell activation, glial cell migration, immune response, phagocytosis, and apoptotic pathways, represent the main functional characteristics of the age 56–60 years as the tipping point of aging.

## 3 Discussion

The advent of high‐throughput sequencing technology has enabled the accumulation of vast and diverse sequencing data in public databases. To conserve resources and maximize the value of existing data, this study aims to collect, organize, and integrate single‐cell sequencing data from public sources focusing on healthy aging of the human brain. The cerebral cortex and hippocampus, are crucial regions related to development, learning, cognition, and neurodegenerative diseases.^[^ ^58^, ^59^, ^60^ ^]^ These regions, compared to others, have more abundant and diverse sequencing data across various age groups. In this study, we integrated data from eight studies conducted over the last five years, depicting single‐cell transcriptome atlas of healthy aging brains from 45 samples. We also provided a comprehensive landscape of cell types and molecular differences between young and aging brains.

We identified 10 major cell types and discovered 1005 genes with age‐related differential expression in specific brain cell types. Notably, several of these genes exhibited age‐related up‐ or downregulation conserved across at least five cell types. Among them, as a sex‐specific gene, XIST was significantly upregulated with aging in specific cell types—including excitatory neurons, inhibitory neurons, and endothelial cells—specifically in female samples, supporting its prior identification as a neural aging marker in the hypothalamus and hippocampus, and highlighting its potential as a female‐specific predictor of neuronal aging.^[^ ^24^ ^]^ At the cell type level, we observed a reduction in the neuronal percentage in the aged group, suggesting neuronal damage or death during aging, while the percentage of glia, including microglia, astrocytes, and oligodendrocytes, significantly increased, indicating their potential roles in aging. Furthermore, we identified key features of brain aging, such as decreased neuronal differentiation, disrupted synaptic assembly and plasticity regulation, and the enrichment of pathways related to neuronal migration inhibition, immune phagocytosis, and apoptosis.

In recent years, the roles of microglia, astrocytes, and oligodendrocytes have gained increasing attention in neurodegenerative diseases and aging.^[^ ^35^, ^61^ ^]^ To explore the association of these three glia with aging, we modeled developmental trajectories for all cell types. As expected,^[^ ^62^, ^63^ ^]^ these glia were located at the later stages of development. Clustering the expression distribution of molecules along the pseudotime revealed that clusters 4, 5, and 6 were primarily involved in synaptogenesis and neural projection development, exhibiting a decline with progression. Cluster 1 was linked to neural and glial cell differentiation and showed a continued rise throughout development. Cluster 2, associated with immune inflammation, was upregulated during the middle and late stages of development before decreasing toward the end. Cluster 3 sharply increased toward the end and primarily inhibited glial cell differentiation. These functional characteristics aligned with age‐related changes, particularly have colocalization with the distribution of the three glia on the trajectory. To further explore the relationship between cell types and aging, we employed SASP gene set scoring, coefficient of variation calculations, and transcriptional noise assessments, revealing that microglia, astrocytes, and oligodendrocytes exhibit higher senescence scores and greater transcriptional instability, and neurons are relatively stable. Our findings emphasize the significant association between glia and age‐related changes in healthy aging brains, suggesting that glial involvement may contribute to impaired neurodevelopment and synaptic regulation during aging.

In several excellent previous studies, different teams of researchers have identified peaks of molecular change waves during aging by DE‐SWAN calculations in plasma proteomics,^[^ ^11^ ^]^ clinical multiomics,^[^ ^13^ ^]^ and multimodal neuroimaging datasets^[^ ^14^ ^]^ at ages 34, 60, and 78, 40, and 60, 57, 70, and 78, respectively. We further applied this method to calculate the molecules that regulate dysregulation at single‐cell resolution, and despite the discrete age distribution of our samples, wave peaks were still identified at 60, 70, and 79 years of age, in general agreement with the results of these previous studies. In addition, the blank in our sample age between 29 and 42 years may have contributed to the lack of observed wave peaks around 40 years of age.

The phenomenon of nonlinear dynamics of human aging found in the above results shares insights with the DNB theory,^[^ ^15^, ^64^, ^65^ ^]^ which suggests that the onset of disease/change in system state is not gradual, but rather a sharp irreversible transition that occurs after reaching a certain tipping point. Therefore, we introduced a DNB computational method to tune the resolution of nonlinear dynamics to the cell‐type level to identify the tipping point of senescence in highly senescence‐associated glia. As a result, we found the brain develops a tipping point at 56–60 years of age that is sharply oriented toward aging, and glia are the first to peak at this age. Neurons, by contrast, exhibit a later tipping point age range, suggesting that they may have relatively slow and passive effects on the critical aging state. We also found tipping points in the brains of 18‐month‐old mice corresponding to the human age group of 56–60 years old, and we have identified several key DNB molecules as candidate genes for aging markers. Further analysis of intercellular communication before and after ages 56–60 also provided insights into the characterization and mechanism of action of tipping points. Notably, our findings on aging tipping points may have broader implications beyond normal aging processes. Given the well‐established association between aging and neurodegenerative diseases, future studies could explore whether these critical transition points are also linked to pathological neurodegeneration, particularly in Alzheimer's disease and related disorders.

In summary, our study emphasizes the importance of nonlinear dynamics characterizing the brain aging process at the single‐cell transcriptome level and pinpoints the resolution of this nonlinear study to the cell type level. More importantly, we introduced the theory of DNB to further identify the critical aging tipping point (around 56–60 years of age) from the nonlinear change, which may be mediated by functional changes in glia. These results provide a new research strategy for the study of brain aging and offer new perspectives on the means of detecting and intervening in brain aging or neurological disorders.

## 4 Experimental Section

### Data Sources

Single‐cell transcriptomics data on the human brain at all ages from adulthood to senescence were collected from eight studies from August 2020 to October 2023.^[^ ^66^, ^67^, ^68^, ^69^, ^70^, ^71^, ^72^, ^73^ ^]^ Data were downloaded from GEO (https://www.ncbi.nlm.nih.gov/geo/) and CELLxGENE (https://cellxgene.cziscience.com/). Data were in a variety of formats including .h5ad, .h5, and .rds. Scanpy (v.1.9.8) in Python (v.3.8.10) as well as Anndata (v.0.7.5.6), Reticulate (v.1.38.0), Seurat (v.4.4.0) in R (v.4.0.2), and Sceasy (v.0.0.7) packages were used for data format conversion to standardize all data formats to .rds for subsequent processing and analysis.

The samples were extracted from autopsy tissues, except for Gerrits2021,^[^ ^69^ ^]^ which was from a donor. To ensure data stability, the selected single‐cell data were subjected to snRNA‐seq (single‐cell nuclear sequencing), and the 10× Genomics platform was used, except for Lu2023,^[^ ^73^ ^]^ which used the self‐developed EasySci‐RNA platform. For all reference datasets, all sample data were excluded from the disease group based on the annotation information provided for each sample. Only the data from the healthy control group were retained for subsequent integrated processing and analysis. Finally, the data consisted of 45 samples from 14 healthy females and 31 healthy males, with an age distribution of 29 to 94 years. The brain regions in the study contain multiple subregions of the cerebral cortex and hippocampus, which have been integrated into two main categories: cerebral cortex and hippocampus. Please refer to the paper (Table S1, Supporting Information) for relevant metadata and detailed information.

### Quality Control and Filtration

Some of the data were processed in Python (v.3.8.10) using Scanpy (v.1.9.8), and most were processed using Seurat (v.4.4.0) in R. Specifically, nuclei with features less than 500 or more than 7500 nuclei were filtered out and nuclei with more than 5% mitochondrial mapping were removed. The final result is a Seurat object of 1 001 088 nuclei and 17 145 genes.

### Integration and Batch Correction of Data

After that the above steps of quality control and filtering the data from each source separately were performed, the CreateSeuratObject function was used to create Seurat objects separately and further merge all the Seurat objects using the Merge function. They were normalized and scaled using NormalizeData and ScaleData functions. Principal component analysis was applied using the RunPCA function and the first 25 components determined based on the ElbowPlot function were used for downstream analysis. To eliminate nonbiological noise due to batch effects, the Harmony algorithm (v.1.2.1) with default parameters was used for batch elimination,^[^ ^19^ ^]^ and downscaling via RunUMAP function after the run.

### Clustering and Identification of Cell Types

Cell clustering analysis used the FindNeighbors and FindClusters functions. DEGs for each cell type were identified with the FindAllMarkers function. The Wilcoxon rank‐sum test was applied to determine the marker genes for each cluster, with criteria of *P*adj < 0.05 and FoldChange > 1.5. Cell types were annotated using CellMarker2.0 (http://117.50.127.228/CellMarker/),^[^ ^20^ ^]^ incorporating classical marker genes identified by various methods.

### Differential Expression Analysis of the Younger and Older Groups

Analysis of differences between cell populations were all identified by DEGs via the FindMarkers function and screened by the Wilcoxon rank‐sum test with *P*adj < 0.05 and FoldChange > 1.2 as the minimum criteria.

### Gene Set Enrichment Analysis

The analysis used the clusterProfiler (v.4.12.6) package in R. The target gene sets were used to identify enrichment pathways or biological processes associated with differentially expressed gene sets. The dataset was derived from the KEGG pathway in the Molecular Signatures Database (MSigDB) (https://www.gsea‐msigdb.org/gsea/index.jsp).^[^ ^74^ ^]^

### Pseudotime Analysis

Pseudotime analysis was performed using the Monocle2 (v.2.32.0) R package. Genes expressed in fewer than 10 cells were first filtered out. Then, the differentialGeneTest function was used to select highly variable genes for sorting, with a significance threshold of *P* < 0.01. The DDRTree algorithm was applied for dimensionality reduction and trajectory construction.

### Gene Set Scoring

Score calculations for specific gene sets were performed using the AUCell (v.1.26.0) R package. AUCell calculates the area under the curve (AUC) to assess whether a critical subset of the input gene set is enriched in expressed genes across each cell. The input expression matrix was first ranked using the AUCell_buildRankings function, followed by the AUCell_calcAUC function to compute the AUC values for subsequent visualization.

### Sample Enrichment Analysis

To assess the enrichment of various cell populations in a sample or phenotype and further explore their relevance, calculations of the relative abundance were performed for each cell population across different samples or phenotypes:

(1) $$E=\frac{n_{A}/N_{A}}{N/N_{total}}$$

where *n*_A_ represents the number of cells of type A in the sample, *N*_A_ is the total number of cells of type A across all samples, and *N* is the total number of cells in the sample. *N*_total_ is the total number of cells across all samples associated with the phenotype. The result, *E*, represents the enrichment score, where *E* > 1 indicates that cell type A is enriched in the sample, and *E* < 1 suggests that the cell type is not relevant to the sample.

### Transcriptional Noise Analysis

Transcriptional noise was quantified as described in previous studies.^[^ ^42^ ^]^ To reduce technical biases due to library size variation, a binomial‐based downsampling approach was applied to equalize UMI counts across cells before the noise calculation. Cell types were categorized into young and old groups, and the corresponding expression matrix was extracted. Genes were ranked based on their mean expression values, and the genes were divided into 10 bins. The first and last bins were excluded. The coefficient of variation for each gene within each bin was calculated, and the genes with the lowest coefficients of variation were selected as conserved genes. This approach aims to select genes with relatively stable expression levels, minimizing technical and biological variability. The mean expression of each conserved gene within each cell type was then used to construct a vector representing that cell type. Finally, the transcriptional noise of each cell was quantified as the Euclidean distance from the cell to the mean of the cell type. A greater distance indicates higher transcriptional noise, reflecting increased cell‐to‐cell variability in gene expression. The Euclidean distances for the young and old groups were compared after statistical tests.

### DE‐SWAN

The DE‐SWAN (v.0.0.0.9001) package was used in R to identify nonlinear wave spikes of molecules during brain aging, based on previous DE‐SWAN.^[^ ^11^ ^]^ For the distribution of the sample over age, 29 sliding window centers were used and the parcel width was set to 20 years for testing. Significance was viewed using *p*‐values less than 0.05 and *q*‐values after the Benjamini–Hochberg correction.

### DNB Analysis

DNB combined with pseudotemporal analysis was used to infer three states of cells: young, tipping point, and old. According to the system dynamics model proposed by DNB theory,^[^ ^15^ ^]^ if a group of genes or DNB modules is found in the gene expression data and this group of genes DNB modules satisfy the following three criteria, then the system is near a tipping point: 1) the standard deviation (SD) of the genes in the group is highly increased; 2) the Pearson correlation coefficient (PCC_in_) of the genes in the group is significantly increased; and 3) the (PCC_out_) between the genes in the group and other groups significantly decreased. Based on these three indices, a composite index (CI) was calculated to be used as the critical score

(2) $$\mathrm{CI}=\frac{\mathrm{PC}C_{i}}{\mathrm{PC}C_{0}}\;\times \;SD_{i}$$

The module with the highest CI score was selected as the dominant group within the resultant cell population, with the genes in this module identified as critical DNB molecules. By comparing the CI scores across different cell populations, the population exhibiting the highest CI is considered to be in a tipping point, indicating that the system is on the brink of undergoing a significant and abrupt transition from one state to another.^[^ ^15^, ^65^ ^]^

### Mfuzz Analytics

The Mfuzz package (v.2.64.0) in R was used for the analysis. This analysis employed the Fuzzy C‐Means Clustering algorithm to model temporal trends and cluster the expression profiles of molecules exhibiting time series characteristics. The input expression matrix was initially created using the new function, followed by normalization using the standardize function. Subsequently, fuzzy clustering was performed with the Mfuzz function, setting the number of clusters to 10 and the fuzzy index to 1.25.^[^ ^75^, ^76^, ^77^ ^]^

### hdWGCNA Analysis

The hdWGCNA analysis was used for weighted gene coexpression network analysis (WGCNA) of single‐cell transcriptome data. Using the hdWGCNA (v.0.3.03) package in R, genes with expression in at least 5% of cells were first selected as input objects, then metacells were constructed using MetacellsByGroups, followed by construction of the coexpression network using the appropriate SoftPower by SetDatExpr and ConstructNetwork functions to construct coexpression networks. For the different modules obtained from clustering, the modules, and age‐related shapes are further correlated using ModuleTraitCorrelation function to see the correlation.

### Analysis of Intercellular Communication

Cell communication and ligand–receptor analyses were performed for three age groups, 50–55, 56–60, and 65–70, using the CellChat (v.1.6.1) software package.^[^ ^78^, ^79^ ^]^ The creation of cell chat objects and the analysis of cell communication networks were completed for each of the three age groups, and then merged for subsequent analysis using the mergeCellChat function, compareInteractions function to calculate the difference between the number and strength of communications, and netVisual_circle function to view the three glia communication networks at different ages, and rankNe function was used to identify conserved and specific pathways at the three ages.

### Gene Set Variation Analysis

The GSVA package (v.1.52.3) in R was used for this analysis. To identify key functional pathways for cells at each age, the mean expression values of marker genes for each age group were used to construct the expression matrix. The gsvaParam function was employed to define the analysis parameters, and gene sets from the Gene Ontology Biological Process in MSigDB were utilized, with a minimum of 5 genes per set. The gsva function was then used to perform enrichment scoring, where the resulting scores reflect the activity level of specific functions at the corresponding age.

### Statistical Analysis

Statistical analyses were performed using R (v4.0.2) and GraphPad Prism (v10.0). Comparisons between groups used unpaired two‐tailed Student's *t*‐test or Wilcoxon rank‐sum test. Differential gene expression considered adjusted *p*‐value < 0.05 and fold change > 1.5 as significant. Enrichment analyses used adjusted *p*‐value < 0.05 as threshold. Data are shown as mean ± SEM. Significance levels are indicated as *P < 0.05, **P < 0.01, ***P < 0.001, and ****P < 0.0001.

## Conflict of Interest

The authors declare no conflict of interest.

## Author Contributions

L.C., R.C.Z., and J.W. conceptualized and designed the study. P.W. performed all the experiments, and wrote the manuscript with feedback from all authors. X.Z. and Z.C. contributed to interpretation of results. Z.C. and J.H. assisted with supplementary analysis. T.D. participated in manuscript revisions and drawings. J.Z. and L.X. assisted in data collection and analysis. L.C. and J.W. critically revised the manuscript. The authors read and approved the final manuscript.

## Supporting information



Supporting Information

Supporting Information

Supporting Information

Supporting Information

Supporting Information

Supporting Information

Supporting Information

Supporting Information

Supporting Information

## Data Availability

The data that support the findings of this study are available from the corresponding author upon reasonable request.

## References

[1] P. Scheltens , B. De Strooper , M. Kivipelto , H. Holstege , G. Chételat , C. E. Teunissen , J. Cummings , W. M. van der Flier , Lancet 2021, 397, 1577. 33667416 10.1016/S0140-6736(20)32205-4 PMC8354300

[2] Y. H. Chou , M. Sundman , V. Ton That , J. Green , C. Trapani , Ageing Res. Rev. 2022, 79, 101660. 35680080 10.1016/j.arr.2022.101660 PMC9707650

[3] E. Tolosa , G. Wenning , W. Poewe , Lancet Neurol. 2006, 5, 75. 16361025 10.1016/S1474-4422(05)70285-4

[4] Y. J. Hou , X. L. Dan , M. Babbar , Y. Wei , S. G. Hasselbalch , D. L. Croteau , V. A. Bohr , Nat. Rev. Neurol. 2019, 15, 565. 31501588 10.1038/s41582-019-0244-7

[5] L. Culig , X. X. Chu , V. A. Bohr , Ageing Res. Rev. 2022, 78, 101636. 35490966 10.1016/j.arr.2022.101636 PMC9168971

[6] T. Bartsch , Eur. J. Neurol. 2017, 24, 742.

[7] S. D. Lee , N. A. Devanney , L. R. Golden , C. T. Smith , J. L. Schwartz , A. E. Walsh , H. A. Clarke , D. S. Goulding , E. J. Allenger , G. Morillo‐Segovia , C. M. Friday , A. A. Gorman , T. R. Hawkinson , S. M. MacLean , H. C. Williams , R. C. Sun , J. M. Morganti , L. A. Johnson , Cell Rep. 42, 112196. 10.1016/j.celrep.2023.112196 PMC10117631 36871219

[8] R. M. Barrientos , M. M. Kitt , L. R. Watkins , S. F. Maier , Neuroscience 2015, 309, 84. 25772789 10.1016/j.neuroscience.2015.03.007 PMC4567963

[9] M. Ximerakis , S. L. Lipnick , B. T. Innes , S. K. Simmons , X. Adiconis , D. Dionne , B. A. Mayweather , L. Nguyen , Z. Niziolek , C. Ozek , V. L. Butty , R. Isserlin , S. M. Buchanan , S. S. Levine , A. Regev , G. D. Bader , J. Z. Levin , L. L. Rubin , Nat. Neurosci. 2019, 22, 1696. 31551601 10.1038/s41593-019-0491-3

[10] J. Rutledge , H. Oh , T. Wyss‐Coray , Nat. Rev. Genet. 2022, 23, 715. 35715611 10.1038/s41576-022-00511-7 PMC10048602

[11] B. Lehallier , D. Gate , N. Schaum , T. Nanasi , S. E. Lee , H. Yousef , P. M. Losada , D. Berdnik , A. Keller , J. Verghese , S. Sathyan , C. Franceschi , S. Milman , N. Barzilai , T. Wyss‐Coray , Nat. Med. 2019, 25, 1843. 31806903 10.1038/s41591-019-0673-2 PMC7062043

[12] J. M. Li , M. Z. Xiong , X. H. Fu , Y. L. Fan , C. Dong , X. Y. Sun , F. Zheng , S. W. Wang , L. X. Liu , M. Xu , C. Wang , J. L. Ping , S. S. Che , Q. R. Wang , K. Yang , Y. S. Zuo , X. Y. Lu , Z. K. Zheng , T. Lan , S. Wang , S. Ma , S. H. Sun , B. Zhang , C. S. Chen , K. Y. Cheng , J. L. Ye , J. Qu , Y. B. Xue , Y. G. Yang , F. Zhang , et al., Med. 2023, 4, 825.37516104

[13] X. Shen , C. Wang , X. Zhou , W. Zhou , D. Hornburg , S. Wu , M. P. Snyder , Nat. Aging 2024, 4, 1619. 39143318 10.1038/s43587-024-00692-2 PMC11564093

[14] W. S. Liu , J. You , S. D. Chen , Y. Zhang , J. F. Feng , Y. M. Xu , J. T. Yu , W. Cheng , Nat. Aging 2025, 5, 99. 39653801 10.1038/s43587-024-00753-6

[15] L. N. Chen , R. Liu , Z. P. Liu , M. Y. Li , K. Aihara , Sci. Rep. 2012, 2, 342. 22461973 10.1038/srep00342 PMC3314989

[16] W. C. Sanderson , S. Scherbov , Demogr. Res. 2007, 16, 27.

[17] K. S. Lee , J. S. Jang , D. R. Lee , Y. H. Kim , G. E. Nam , B. D. Han , K. D. Han , K. H. Cho , S. M. Kim , Y. S. Choi , D. H. Kim , J. Bone Miner. Metab. 2014, 32, 683. 24337956 10.1007/s00774-013-0540-z

[18] A. A. Wawer , A. Jennings , S. J. Fairweather‐Tait , Mech. Ageing Dev. 2018, 175, 55. 30040993 10.1016/j.mad.2018.07.003

[19] I. Korsunsky , N. Millard , J. Fan , K. Slowikowski , F. Zhang , K. Wei , Y. Baglaenko , M. Brenner , P. R. Loh , S. Raychaudhuri , Nat. Methods 2019, 16, 1289. 31740819 10.1038/s41592-019-0619-0 PMC6884693

[20] C. X. Hu , T. Y. Li , Y. Q. Xu , X. X. Zhang , F. Li , J. Bai , J. Chen , W. Q. Jiang , K. Y. Yang , Q. Ou , X. Li , P. Wang , Y. P. Zhang , Nucleic Acids Res. 2023, 51, D870. 36300619 10.1093/nar/gkac947 PMC9825416

[21] Y. J. Su , Y. Zhou , M. L. Bennett , S. Y. Li , M. Carceles‐Cordon , L. Lu , S. Huh , D. Jimenez‐Cyrus , B. C. Kennedy , S. K. Kessler , A. N. Viaene , I. Helbig , X. S. Gu , J. E. Kleinman , T. M. Hyde , D. R. Weinberger , D. W. Nauen , H. J. Song , G. L. Ming , Cell Stem Cell 2022, 29, 1594. 36332572 10.1016/j.stem.2022.09.010 PMC9844262

[22] S. J. Zhong , W. Y. Ding , L. Sun , Y. F. Lu , H. Dong , X. Y. Fan , Z. Y. Liu , R. G. Chen , S. Zhang , Q. Ma , F. C. Tang , Q. Wu , X. Q. Wang , Nature 2020, 577, 531. 31942070 10.1038/s41586-019-1917-5

[23] X. Chen , Y. Huang , L. Huang , Z. Huang , Z. Z. Hao , L. Xu , N. Xu , Z. Li , Y. Mou , M. Ye , R. You , X. Zhang , S. Liu , Z. Miao , Nat. Med. 2024, 30, 2679. 39095595 10.1038/s41591-024-03150-z PMC11405287

[24] K. H. Hajdarovic , D. D. Yu , L. A. Hassell , S. A. Evans , S. Packer , N. Neretti , A. E. Webb , Nat. Aging 2022, 2, 662. 36285248 10.1038/s43587-022-00246-4 PMC9592060

[25] T. L. Kremer , J. Chen , A. Buhl , O. Berhe , E. Bilek , L. S. Geiger , R. Ma , C. Moessnang , M. Reichert , I. Reinhard , K. Schwarz , J. I. Schweiger , F. Streit , S. H. Witt , Z. Zang , X. Zhang , M. M. Nöthen , M. Rietschel , U. W. Ebner‐Priemer , E. Schwarz , A. Meyer‐Lindenberg , U. Braun , H. Tost , Biol. Psychiatry 2024, 96, 858. 38460581 10.1016/j.biopsych.2024.03.003

[26] C. Zimmer , H. E. Hanson , D. E. Wildman , M. Uddin , L. B. Martin , BioScience 2020, 70, 1127.

[27] A. S. Zannas , M. W. Jia , K. Hafner , J. Baumert , T. Wiechmann , J. C. Pape , J. Arloth , M. Ködel , S. Martinelli , M. Roitman , S. Röh , A. Haehle , R. T. Emeny , S. Iurato , T. Carrillo‐Roa , J. Lahti , K. Räikkönen , J. G. Eriksson , A. J. Drake , M. Waldenberger , S. Wahl , S. Kunze , S. Lucae , B. Bradley , C. Gieger , F. Hausch , A. K. Smith , K. J. Ressler , B. Müller‐Myhsok , K. H. Ladwig , et al., Proc. Natl. Acad. Sci. USA 2019, 116, 11370. 31113877 10.1073/pnas.1816847116 PMC6561294

[28] C. J. Kearney , K. L. Randall , J. Oliaro , Cell Mol. Immunol. 2017, 14, 406. 28366940 10.1038/cmi.2017.9 PMC5423093

[29] J. Englund , J. Haikonen , V. Shteinikov , S. P. Amarilla , T. Atanasova , A. Shintyapina , M. Ryazantseva , J. Partanen , V. Voikar , S. E. Lauri , Transl. Psychiatry 2021, 11. 10.1038/s41398-021-01654-7 PMC8523542 34663781

[30] M. E. Williams , S. A. Wilke , A. Daggett , E. Davis , S. Otto , D. Ravi , B. Ripley , E. A. Bushong , M. H. Ellisman , G. Klein , A. Ghosh , Neuron 2011, 71, 640. 21867881 10.1016/j.neuron.2011.06.019 PMC3272880

[31] C. S. Jayasena , M. E. Bronner , J. Cell Biol. 2012, 199, 453. 23091072 10.1083/jcb.201204138 PMC3483135

[32] R. Bartolomeo , L. Cinque , C. De Leonibus , A. Forrester , A. C. Salzano , J. Monfregola , E. De Gennaro , E. Nusco , I. Azario , C. Lanzara , M. Serafini , B. Levine , A. Ballabio , C. Settembre , J. Clin. Invest. 2017, 127, 3717. 28872463 10.1172/JCI94130 PMC5617676

[33] S. Dikiy , J. Li , L. Bai , M. L. Jiang , L. Janke , X. Y. Zong , X. L. Hao , B. Hoyos , Z. M. Wang , B. S. Xu , Y. P. Fan , A. Y. Rudensky , Y. Q. Feng , Immunity 2021, 54, 931. 33838102 10.1016/j.immuni.2021.03.020 PMC8317508

[34] C. Cserep , A. D. Schwarcz , B. Posfai , Z. I. Laszlo , A. Kellermayer , Z. Koernyei , M. Kisfali , M. Nyerges , Z. Lele , I. Katona , A. Denes , Cell Rep. 2022, 40, 111369. 36130488 10.1016/j.celrep.2022.111369 PMC9513806

[35] N. N. Fancy , A. M. Smith , A. Caramello , S. Tsartsalis , K. Davey , R. C. J. Muirhead , A. Mcgarry , M. H. Jenkyns , E. Schneegans , V. Chau , M. Thomas , S. Boulger , T. K. D. Cheung , E. Adair , M. Papageorgopoulou , N. Willumsen , C. Khozoie , D. Gomez‐Nicola , J. S. Jackson , P. M. Matthews , Acta Neuropathol. 2024, 147, 78. 38695952 10.1007/s00401-024-02727-9 PMC11065703

[36] S. Pandey , K. Shen , S. H. Lee , Y. A. A. Shen , Y. Y. Wang , M. Otero‐García , N. Kotova , S. T. Vito , B. Laufer , D. F. Newton , M. G. Rezzonico , J. E. Hanson , J. S. Kaminker , C. J. Bohlen , T. J. Yuen , B. A. Friedman , Cell Rep. 2022, 40, 111189. 36001972 10.1016/j.celrep.2022.111189

[37] M. Linnerbauer , M. A. Wheeler , F. J. Quintana , Neuron 2020, 108, 608. 32898475 10.1016/j.neuron.2020.08.012 PMC7704785

[38] M. W. Salter , B. Stevens , Nat. Med. 2017, 23, 1018. 28886007 10.1038/nm.4397

[39] B. S. Wang , J. Han , J. H. Elisseeff , M. Demaria , Nat. Rev. Mol. Cell Biol. 2024, 25, 958. 38654098 10.1038/s41580-024-00727-x

[40] S. K. Dehkordi , J. Walker , E. Sah , E. Bennett , F. Atrian , B. Frost , B. Woost , R. E. Bennett , T. C. Orr , Y. Y. Zhou , P. S. Andhey , M. Colonna , P. H. Sudmant , P. Xu , M. H. Wang , B. Zhang , H. Zare , M. E. Orr , Nat. Aging 2021, 1, 1107. 35531351 10.1038/s43587-021-00142-3 PMC9075501

[41] C. P. Martinez‐Jimenez , N. Eling , H. C. Chen , C. A. Vallejos , A. A. Kolodziejczyk , F. Connor , L. Stojic , T. F. Rayner , M. J. T. Stubbington , S. A. Teichmann , M. de la Roche , J. C. Marioni , D. T. Odom , Science 2017, 355, 1433. 28360329 10.1126/science.aah4115 PMC5405862

[42] I. Angelidis , L. M. Simon , I. E. Fernandez , M. Strunz , C. H. Mayr , F. R. Greiffo , G. Tsitsiridis , M. Ansari , E. Graf , T. M. Strom , M. Nagendran , T. Desai , O. Eickelberg , M. Mann , F. J. Theis , H. B. Schiller , Nat. Commun. 2019, 10, 963. 30814501 10.1038/s41467-019-08831-9 PMC6393476

[43] H. Zhang , J. M. Li , J. Ren , S. H. Sun , S. Ma , W. Q. Zhang , Y. Yu , Y. S. Cai , K. W. Yan , W. Li , B. Y. Hu , P. Chan , G. G. Zhao , J. C. I. Belmonte , Q. Zhou , J. Qu , S. Wang , G. H. Liu , Protein Cell 2021, 12, 695. 34052996 10.1007/s13238-021-00852-9 PMC8403220

[44] H. Y. Huang , J. R. Bach , H. S. Sharma , H. Saberi , S. R. Jeon , X. L. Guo , A. Shetty , Z. Hawamdeh , A. Sharma , K. von Wild , D. Siniscalco , P. R. Sanberg , Y. Hu , M. Z. Xue , L. Chen , F. B. Han , A. Otom , J. Z. Hu , Q. Q. Zhang , J. Neurorestoratol. 2023, 11, 100054.

[45] T. Vellai , K. Takacs‐Vellai , Y. Zhang , A. L. Kovacs , L. Orosz , F. Müller , Nature 2003, 426, 620. 10.1038/426620a 14668850

[46] V. Wanke , E. Cameroni , A. Uotila , M. Piccolis , J. Urban , R. Loewith , C. De Virgilio , Mol. Microbiol. 2008, 69, 277. 18513215 10.1111/j.1365-2958.2008.06292.x

[47] D. E. Harrison , R. Strong , Z. D. Sharp , J. F. Nelson , C. M. Astle , K. Flurkey , N. L. Nadon , J. E. Wilkinson , K. Frenkel , C. S. Carter , M. Pahor , M. A. Javors , E. Fernandez , R. A. Miller , Nature 2009, 460, 392. 19587680 10.1038/nature08221 PMC2786175

[48] M. Xu , T. Tchkonia , H. Ding , M. Ogrodnik , E. R. Lubbers , T. Pirtskhalava , T. A. White , K. O. Johnson , M. B. Stout , V. Mezera , N. Giorgadze , M. D. Jensen , N. K. LeBrasseur , J. L. Kirkland , Proc. Natl. Acad. Sci. USA 2015, 112, 6301. 26578790 10.1073/pnas.1515386112 PMC4655580

[49] M. Xu , A. K. Palmer , H. Ding , M. M. Weivoda , T. Pirtskhalava , T. A. White , A. Sepe , K. O. Johnson , M. B. Stout , N. Giorgadze , M. D. Jensen , N. K. LeBrasseur , T. Tchkonia , J. L. Kirkland , eLife 2015, 4, 12997. 10.7554/eLife.12997 PMC4758946 26687007

[50] S. Nanda , A. Calderon , A. Sachan , T. T. Duong , J. Koch , X. Xin , D. Solouk‐Stahlberg , Y. W. Wu , P. Nalbant , L. Dehmelt , Nat. Commun. 2023, 14, 8356. 38102112 10.1038/s41467-023-43875-y PMC10724141

[51] J. Du , Z. Zhu , L. Xu , X. Chen , X. Li , T. Lan , W. Li , K. Yuan , Y. Zeng , Aging 2020, 12, 20235. 33122451 10.18632/aging.103772 PMC7655160

[52] K. R. Groot , L. M. Sevilla , K. Nishi , T. DiColandrea , F. M. Watt , J. Cell Biol. 2004, 166, 653. 15337775 10.1083/jcb.200312123 PMC2172441

[53] O. Hahn , A. G. Foltz , M. Atkins , B. Kedir , P. Moran‐Losada , I. H. Guldner , C. Munson , F. Kern , R. Palovics , N. Lu , H. Zhang , A. Kaur , J. Hull , J. R. Huguenard , S. Gronke , B. Lehallier , L. Partridge , A. Keller , T. Wyss‐Coray , Cell 2023, 186, 4117. 37591239 10.1016/j.cell.2023.07.027 PMC10528304

[54] R. C. Meyer , M. M. Giddens , S. A. Schaefer , R. A. Hall , Proc. Natl. Acad. Sci. USA 2013, 110, 9529. 23690594 10.1073/pnas.1219004110 PMC3677493

[55] M. S. Subbarayan , A. Joly‐Amado , P. C. Bickford , K. R. Nash , Pharmacol. Ther. 2022, 231, 107989. 34492237 10.1016/j.pharmthera.2021.107989

[56] S. Wu , R. Xue , S. Hassan , T. M. L. Nguyen , T. Wang , H. Pan , J. Xu , Q. Liu , W. Zhang , Z. Wen , Dev. Cell 2018, 46, 552. 30205037 10.1016/j.devcel.2018.08.005

[57] J. H. Zhao , Q. Li , X. T. Ouyang , F. Wang , Q. Li , Z. X. Xu , D. X. Ji , Q. W. Wu , J. Zhang , C. B. Lu , S. B. Ji , S. M. Li , J. Neurorestoratol. 2023, 11, 100042.

[58] N. P. Friedman , T. W. Robbins , Neuropsychopharmacology 2022, 47, 72.34408280

[59] C. Amiez , C. Verstraete , J. Sallet , F. Hadj‐Bouziane , S. Ben Hamed , A. Meguerditchian , E. Procyk , C. R. E. Wilson , M. Petrides , C. C. Sherwood , W. D. Hopkins , Commun. Biol. 2023, 6, 693. 37407769 10.1038/s42003-023-05066-9 PMC10322890

[60] T. Bartsch , P. Wulff , Neuroscience 2015, 309, 1. 26241337 10.1016/j.neuroscience.2015.07.084

[61] W. E. Allen , T. R. Blosser , Z. A. Sullivan , C. Dulac , X. W. Zhuang , Cell 2023, 186, 194. 36580914 10.1016/j.cell.2022.12.010 PMC10024607

[62] J. L. Li , F. H. Yang , Y. Tian , Z. W. Wang , D. S. Qi , Z. G. Yang , J. G. Song , J. Ding , X. Wang , Z. Z. Zhang , eLife 2024, 11, RP94317.

[63] L. Yang , Z. M. Y. Li , G. P. Liu , X. S. Li , Z. G. Yang , Neurosci. Bull. 2022, 38, 47. 34374948 10.1007/s12264-021-00759-9 PMC8783027

[64] B. W. Yang , M. Y. Li , W. Q. Tang , W. X. Liu , S. Zhang , L. N. Chen , J. L. Xia , Nat. Commun. 2018, 9, 678. 29445139 10.1038/s41467-018-03024-2 PMC5813207

[65] Z. Y. Fang , X. K. Han , Y. Q. Chen , X. Y. Tong , Y. Xue , S. Yao , S. J. Tang , Y. J. Pan , Y. H. Sun , X. Wang , Y. J. Jin , H. Q. Chen , L. Hu , L. J. Hui , L. Li , L. N. Chen , H. B. Ji , Signal Transduction Targeted Ther. 2023, 8, 16. 10.1038/s41392-022-01227-0 PMC9832009 36627278

[66] K. Siletti , R. Hodge , A. M. Albiach , K. W. Lee , S. L. Ding , L. J. Hu , P. Lönnerberg , T. Bakken , T. Casper , M. Clark , N. Dee , J. Gloe , D. Hirschstein , N. V. Shapovalova , C. D. Keene , J. Nyhus , H. Tung , A. M. Yanny , E. Arenas , E. S. Lein , S. Linnarsson , Science 2023, 382, 175. 10.1126/science.add7046 37824663

[67] S. F. Lau , H. Cao , A. K. Y. Fu , N. Y. Ip , Proc. Natl. Acad. Sci. USA 2020, 117, 25800. 32989152 10.1073/pnas.2008762117 PMC7568283

[68] A. M. Smith , K. Davey , S. Tsartsalis , C. Khozoie , N. Fancy , S. S. Tang , E. Liaptsi , M. Weinert , A. McGarry , R. C. J. Muirhead , S. Gentleman , D. R. Owen , P. M. Matthews , Acta Neuropathol. 2022, 143, 75. 34767070 10.1007/s00401-021-02372-6 PMC8732962

[69] E. Gerrits , N. Brouwer , S. M. Kooistra , M. E. Woodbury , Y. Vermeiren , M. Lambourne , J. Mulder , M. Kummer , T. Möller , K. Biber , W. Dunnen , P. P. De Deyn , B. J. L. Eggen , E. Boddeke , Acta Neuropathol. 2021, 141, 681. 33609158 10.1007/s00401-021-02263-w PMC8043951

[70] D. Agarwal , C. Sandor , V. Volpato , T. M. Caffrey , J. Monzón‐Sandoval , R. Bowden , J. Alegre‐Abarrategui , R. Wade‐Martins , C. Webber , Nat. Commun. 2020, 11, 4183. 32826893 10.1038/s41467-020-17876-0 PMC7442652

[71] W. Wang , M. Wang , M. Yang , B. Zeng , W. Qiu , Q. Ma , X. Jing , Q. Zhang , B. Wang , C. Yin , J. Zhang , Y. Ge , Y. Lu , W. Ji , Q. Wu , C. Ma , X. Wang , Cell Res. 2022, 32, 729. 35750757 10.1038/s41422-022-00678-y PMC9343414

[72] D. Franjic , M. Skarica , S. Ma , J. I. Arellano , A. T. N. Tebbenkamp , J. Choi , C. Xu , Q. Li , Y. M. Morozov , D. Andrijevic , Z. Vrselja , A. Spajic , G. Santpere , M. Li , S. Zhang , Y. Liu , J. Spurrier , L. Zhang , I. Gudelj , L. Rapan , H. Takahashi , A. Huttner , R. Fan , S. M. Strittmatter , A. M. M. Sousa , P. Rakic , N. Sestan , Neuron 2022, 110, 452. 34798047 10.1016/j.neuron.2021.10.036 PMC8813897

[73] Z. Lu , M. Zhang , J. Lee , A. Sziraki , S. Anderson , Z. Zhang , Z. Xu , W. Jiang , S. Ge , P. T. Nelson , W. Zhou , J. Cao , Cell 2023, 186, 4345. 37774676 10.1016/j.cell.2023.08.042 PMC10545416

[74] A. Liberzon , A. Subramanian , R. Pinchback , H. Thorvaldsdóttir , P. Tamayo , J. P. Mesirov , M. database , Bioinformatics 2011, 27, 1739. 21546393 10.1093/bioinformatics/btr260 PMC3106198

[75] L. Kumar , E. F. M. Mfuzz , Bioinformation 2007, 2, 5. 18084642 10.6026/97320630002005 PMC2139991

[76] J. Oberstaller , S. J. Joseph , J. C. Kissinger , BMC Genomics 2013, 14, 516. 23895416 10.1186/1471-2164-14-516 PMC3734150

[77] B. Yang , M. Li , W. Tang , W. Liu , S. Zhang , L. Chen , J. Xia , Nat. Commun. 2018, 9, 678. 29445139 10.1038/s41467-018-03024-2 PMC5813207

[78] S. Jin , C. F. Guerrero‐Juarez , L. Zhang , I. Chang , R. Ramos , C. H. Kuan , P. Myung , M. V. Plikus , Q. Nie , Nat. Commun. 2021, 12, 1088. 33597522 10.1038/s41467-021-21246-9 PMC7889871

[79] S. Jin , M. V. Plikus , Q. Nie , Nat. Protoc. 2025, 20, 180. 39289562 10.1038/s41596-024-01045-4

