# Supplementary material for: Identifying Tipping Points during Healthy Brain Aging through Single‐Nucleus Transcriptomic Analysis
Source: Adv Sci (Weinh). 2025 Aug 19;12(41):e05779. doi: 10.1002/advs.202505779 (PMC12591157; doi:10.1002/advs.202505779)
Supplement: Supplementary file 1 — Supporting Information [file ADVS-12-e05779-s008.docx]

**Supporting Information**

**Identifying Tipping Points During Healthy Brain Aging Through Single-Nucleus Transcriptomic Analysis**

Peiru Wu, Xuyu Zhao, Zixin Chen, Jingying Huang, Tengteng Dai, Jianxin Zhou, Luyao Xiao, Luonan Chen^*^, Robert Chunhua Zhao^*^, and Jiao Wang^*^


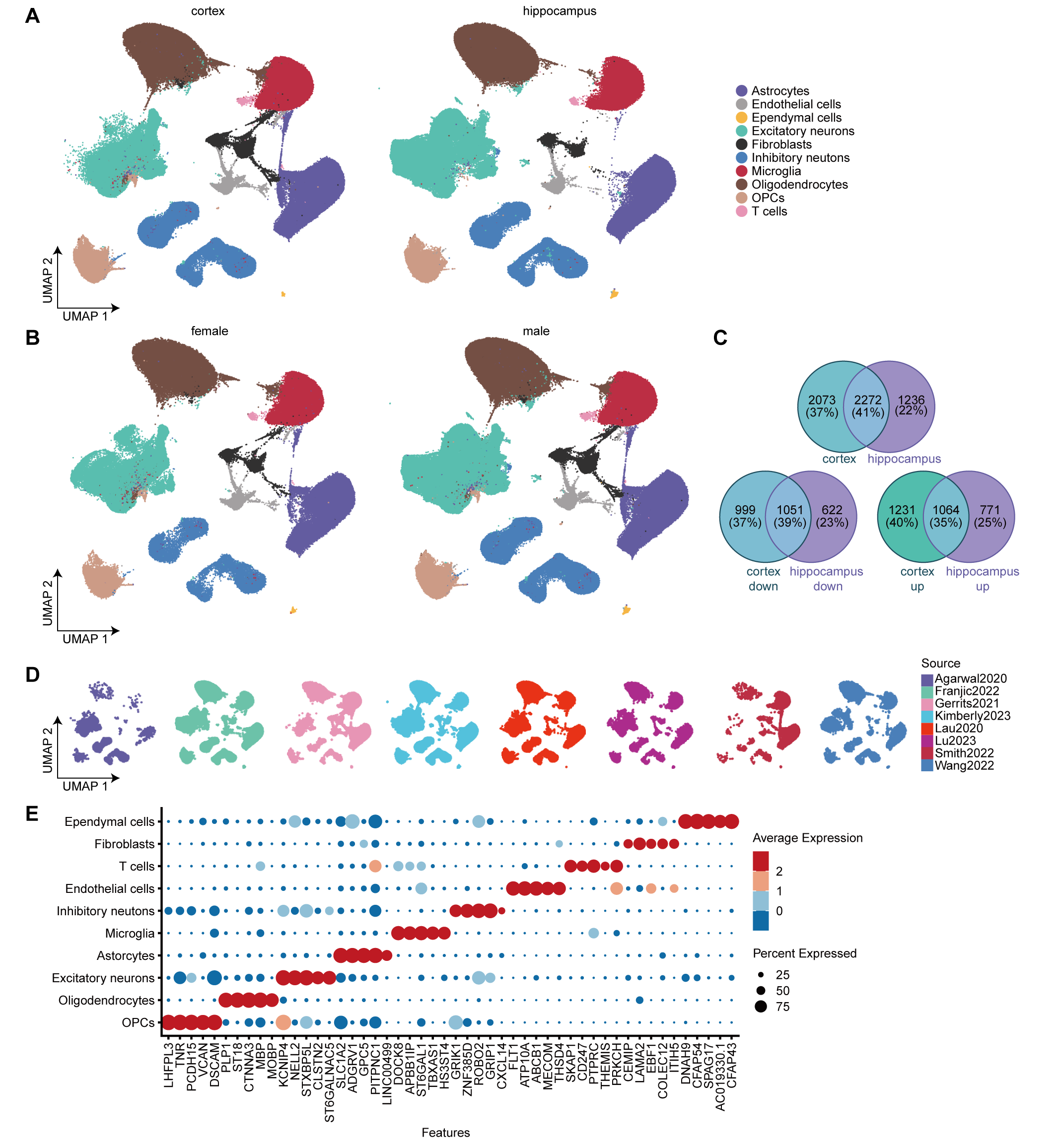


**Figure S1.** UMAP and markers for cell types in different samples. A,B) UMAP of the distribution of different cell types in different brain tissues (A) and in different sexes (B). (Sample: cortex *N* = 29; hippocampus *N* = 20; female *N* = 14; male *N* = 31; Cellnum: cortex *n* = 567798; hippocampus *n* = 433290; female *n* = 174299; male *n* = 826789). C) Consistency of molecular differential expression in the cortex vs. hippocampus during aging. (*P*adj < 0.05, log2FoldChange > 0.58). D) UMAP presentation for 8 data sources after batch effect removal. E) Different cell types sorted top5 markers by Foldchange (*P*adj < 0.05, FoldChange > 1.5).

**
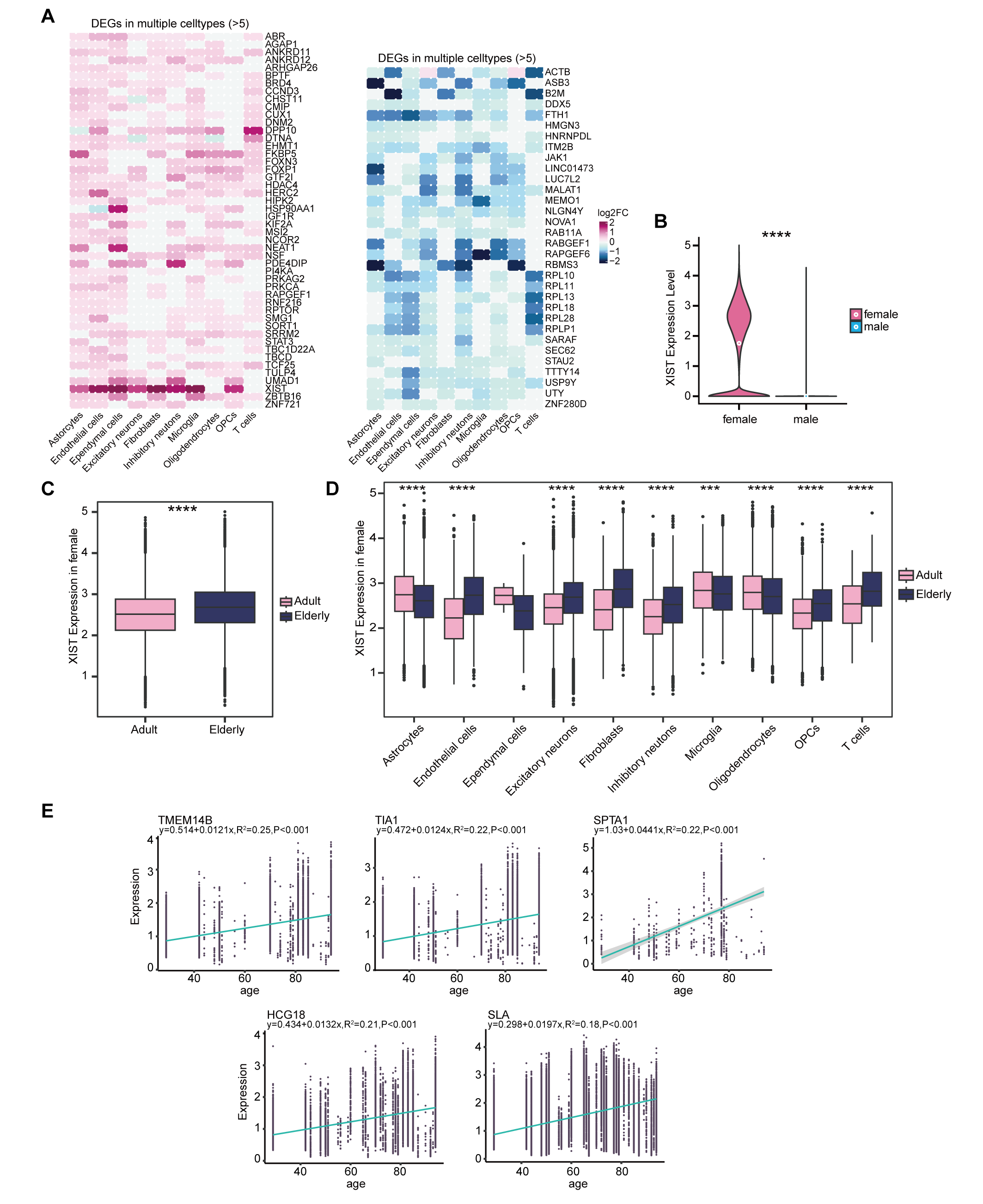
**

**Figure S2.** Differential changes and nonlinear features of molecules in aging. A) Genes differentially up- or down-regulated in expression in more than 5 cell types (*P*adj < 0.05, log2FoldChange > 0.25). B) XIST gene expression in sex groups. C,D) Differential expression of the XIST gene in female samples at the overall (C), and cell type level (D) in young and old groups. (Wilcoxon rank-sum test, ^*^*P* < 0.05,^**^*P* < 0.01,^***^*P* < 0.001,^****^*P* < 0.0001). E) Linear regression analysis revealed a nonlinear trend in gene changes with age. The expression of each gene was obtained from each cell in the samples of each age, and the top5 genes were displayed in order of correlation coefficient.


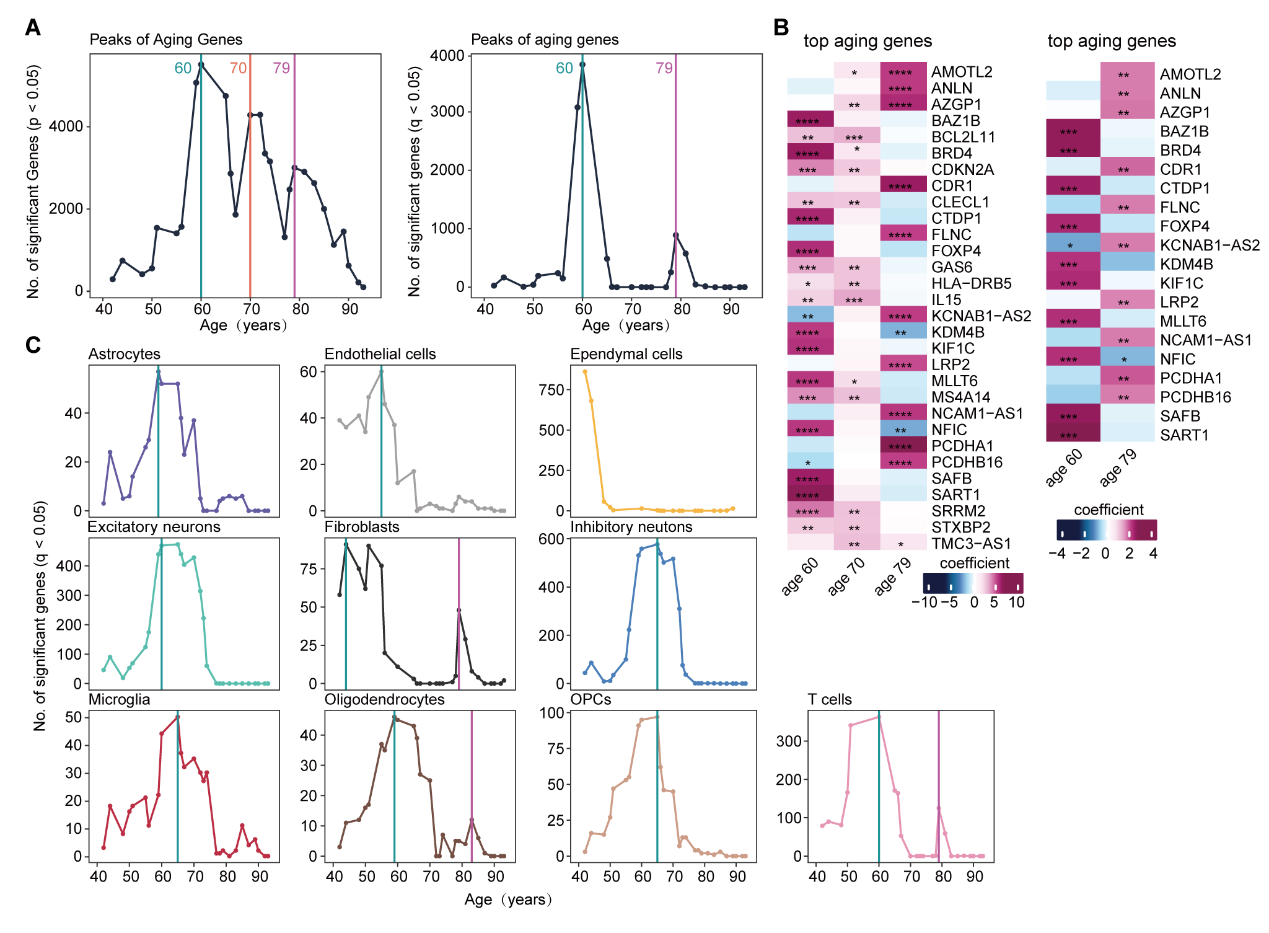


**Figure S3.** Waves of destabilizing molecular changes in aging. A) Number of genes with erratic changes in aging, identified peaks at 60, 70 and 79 years of age (*P*_(70)_ < 0.05, *q*_(60,79)_ < 0.05). B) Top genes identified by DE-SWAN at peak age, red and blue colors represent the increase and decrease of genes at that age, respectively (**P/q* < 0.05,***P/q* < 0.01,****P/q* < 0.001,*****P/q* < 0.0001). C) Wave peaks of genes in each cell type (*q* < 0.05).


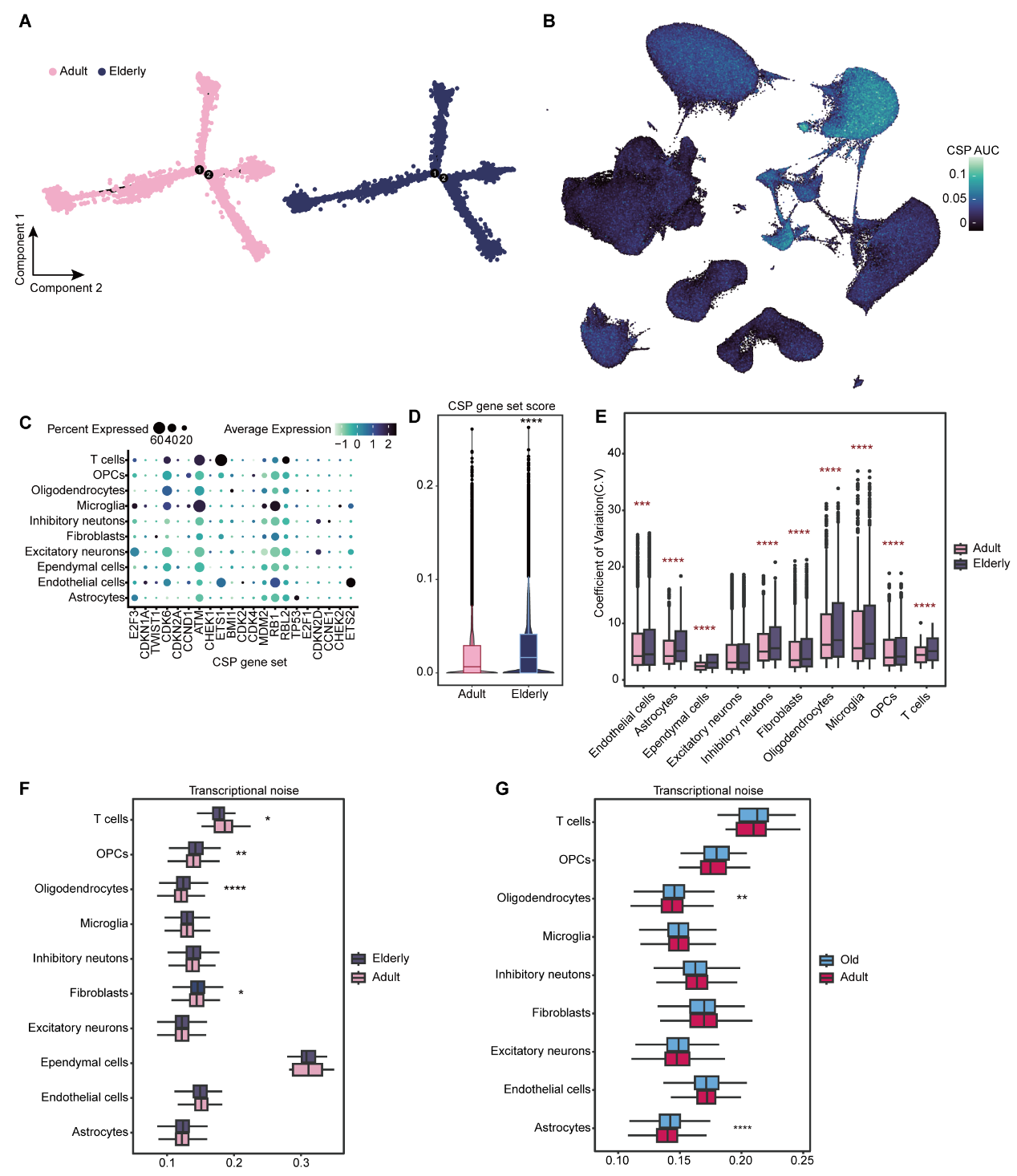


**Figure S4.** High correlation between glia and aging. A) Distribution of Adult and elderly samples over pseudotime trajectories. B) Canonical senescence phenotype (CSP) gene set scoring shows microglia have the highest score. C) CSP gene expression in different cell types. D) CSP scores were significantly higher in the elderly group than in the adult group. E) Coefficient of variation calculations showed generally higher coefficients of variation in the elderly group than in the adult group for each cell type. F,G) Transcriptional noise calculations demonstrating noise differences between the elderly and adult groups (F), and the adult and old groups (ages 29-60 and 60-74) (G) (Wilcoxon rank-sum test, ^*^*P* < 0.05,^**^*P* < 0.01,^***^*P* < 0.001,^****^*P* < 0.0001).


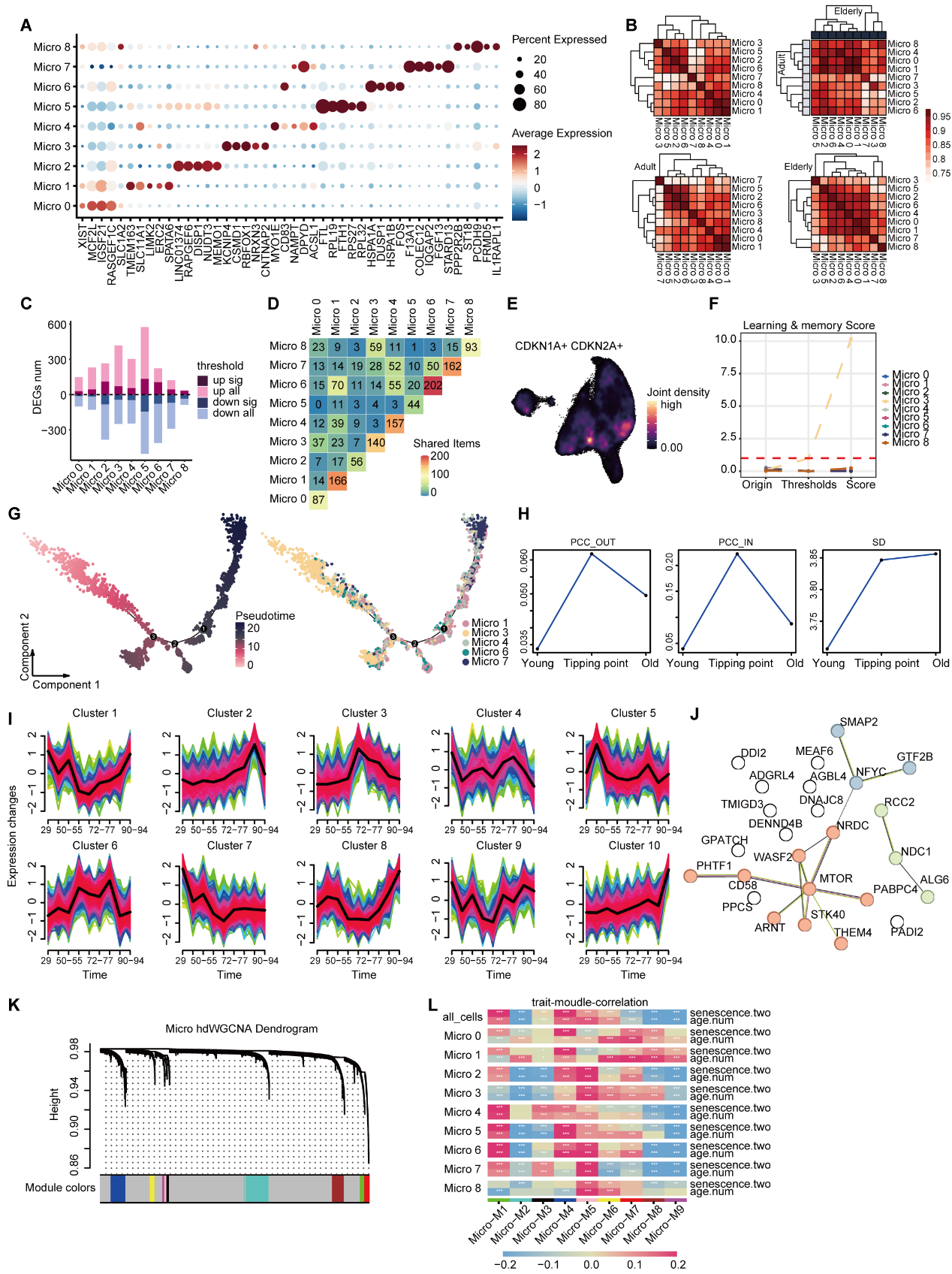


**Figure S5.** Identification of nonlinear critical states in microglia during senescence. A) Markers for each subtype identified in microglia. B) Spearman correlation is used to see the correlation of different cell types being in different age groups. C,D) Analysis of differences between microglia subtypes. The number of differential genes by subtype is shown, with light colors Foldchange > 1.2,dark colors indicating the number of genes with Foldchange > 1.5 (C), and the number of intersections between GO-enriched term of each subtype (D). E) Enrichment of senescence markers CDKN1A (p21) and CDKN2A (p16) in microglial cells. F) The scoring results for the set of genes related to learning and memory functions showed that Micro3 has a very high score. G) Distribution of senescence-associated isoforms on pseudotime trajectories. H) Three key criteria in the DNB composite index (CI) calculation. I) Clustering of expression trends of all genes in microglia at different ages by Mfuzz reflects the nonlinearity of molecules in aging. J) Key DNB molecular networks at tipping point. K,L) Clustering of the nine modules obtained from the hdWGCNA analysis (K) and the correlation between each module and the age-related shape (L) (Student's t-test, ^*^*q* < 0.05,^**^*q* < 0.01,^***^*q* < 0.001,^****^*q* < 0.0001).


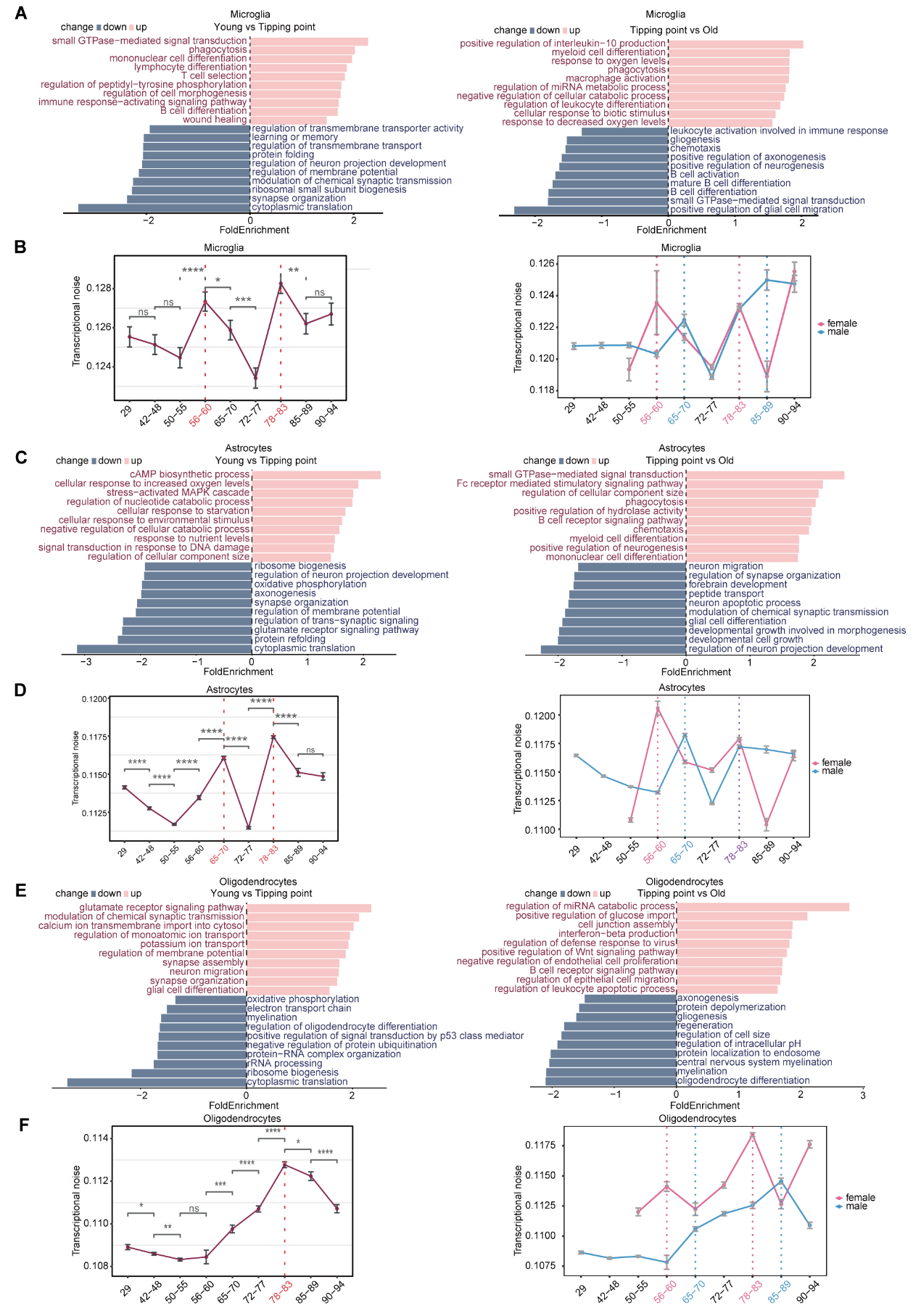


**Figure S6.** Tipping point functional characterization and peak transcriptional noise in three kinds of glia. A,C,E) Functional differences between the respective tipping point cells of microglia astrocytes and oligodendrocytes and the other two DNB state cells (P adj < 0.05). B,D,F) Transcriptional noise calculations show peak noise for microglia astrocytes and oligodendrocytes at specific age stages (Wilcoxon rank-sum test, ^*^*P* < 0.05,^**^*P* < 0.01,^***^*P* < 0.001,^****^*P* < 0.0001).


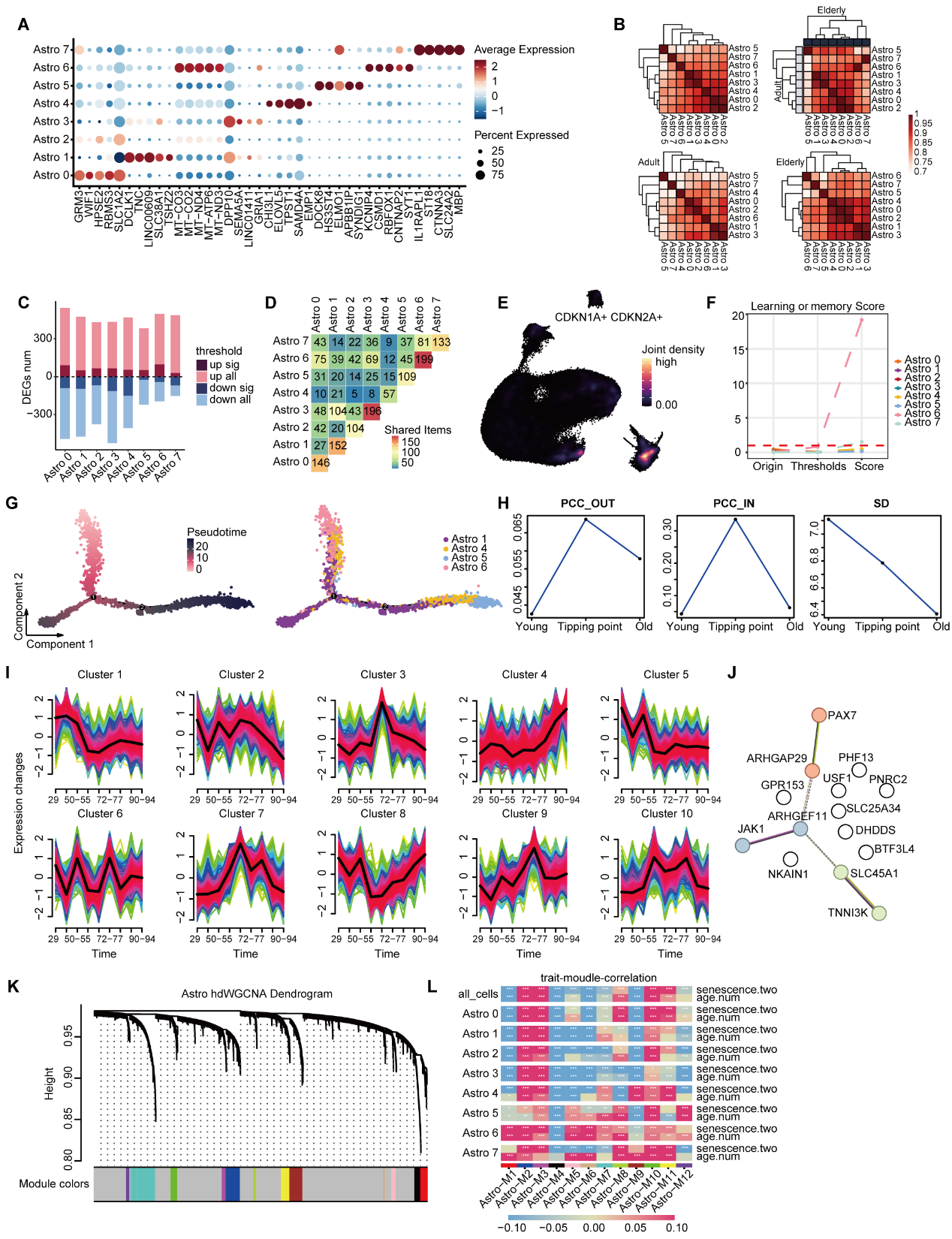


**Figure S7.** Identification of nonlinear critical states in astrocytes during senescence. A) Markers for each subtype identified in astrocytes. B) Spearman correlation is used to see the correlation of different cell types being in different age groups. C,D) Analysis of differences between astrocytes subtypes. The number of differential genes by subtype is shown, with light colors Foldchange > 1.2,dark colors indicating the number of genes with Foldchange > 1.5 (C), and the number of intersections between GO-enriched term of each subtype (D). E) Enrichment of senescence markers CDKN1A (p21) and CDKN2A (p16) in astrocytes cells. F) The scoring results for the set of genes related to learning and memory functions showed that Astro6 has a very high score. G) Distribution of senescence-associated isoforms on pseudotime trajectories. H) Three key criteria in the DNB composite index (CI) calculation. I) Clustering of expression trends of all genes in astrocytes at different ages by Mfuzz reflects the nonlinearity of molecules in aging. J) Key DNB molecular networks at tipping point. K,L) Clustering of the nine modules obtained from the hdWGCNA analysis (K) and the correlation between each module and the age-related shape (L) (Student's t-test, ^*^*q* < 0.05,^**^*q* < 0.01,^***^*q* < 0.001,^****^*q* < 0.0001).


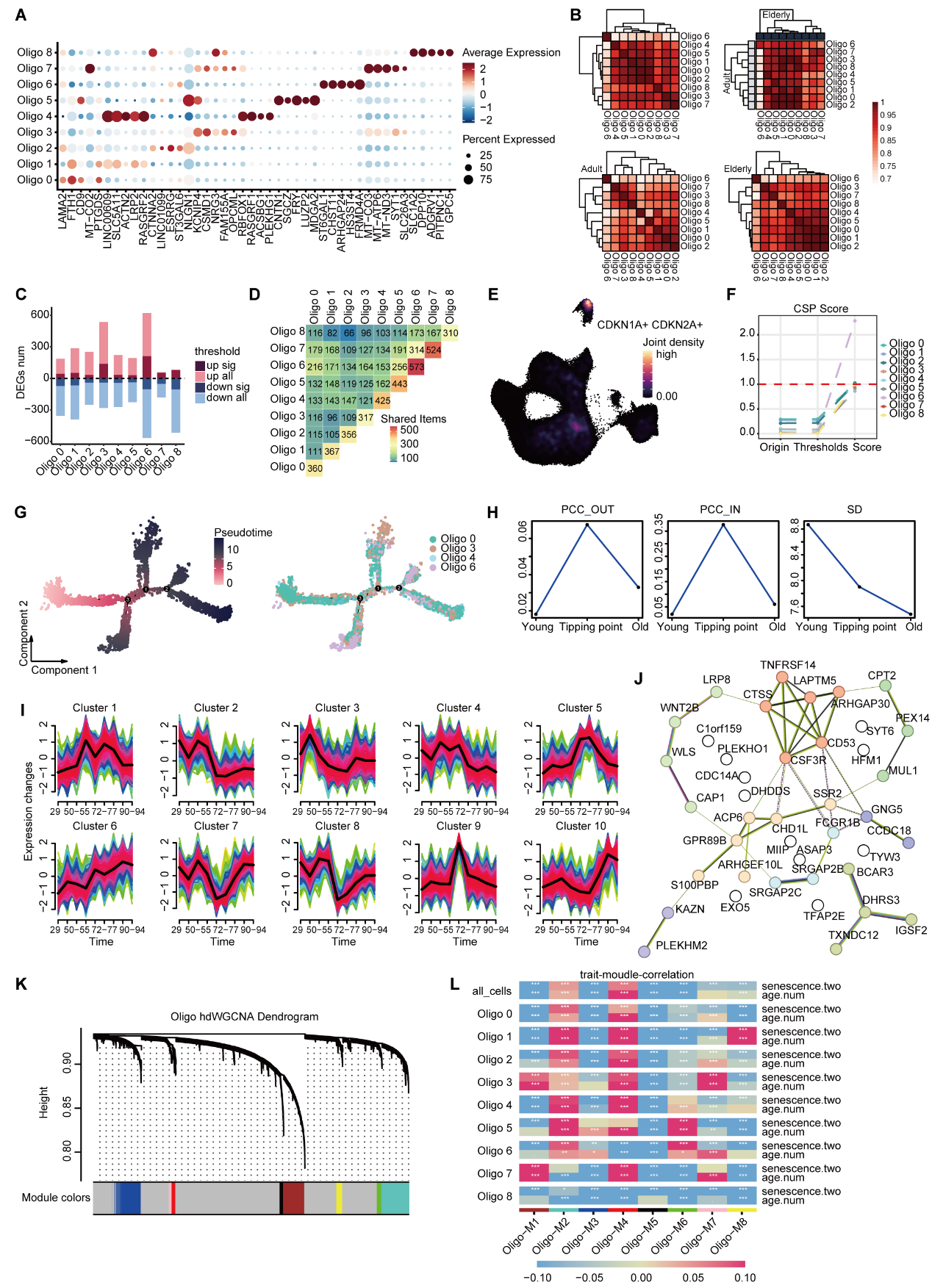


**Figure S8.** Identification of nonlinear critical states in oligodendrocytes during senescence. A) Markers for each subtype identified in oligodendrocytes. B) Spearman correlation is used to see the correlation of different cell types being in different age groups. C,D) Analysis of differences between oligodendrocytes subtypes. The number of differential genes by subtype is shown, with light colors Foldchange > 1.2,dark colors indicating the number of genes with Foldchange > 1.5 (C), and the number of intersections between GO-enriched term of each subtype (D). E) Enrichment of senescence markers CDKN1A (p21) and CDKN2A (p16) in oligodendrocytes cells. F) The scoring results for the set of genes related to CSP. G) Distribution of senescence-associated isoforms on pseudotime trajectories. H) Three key criteria in the DNB composite index (CI) calculation. I) Clustering of expression trends of all genes in oligodendrocytes at different ages by Mfuzz reflects the nonlinearity of molecules in aging. J) Key DNB molecular networks at tipping point. K,L) Clustering of the nine modules obtained from the hdWGCNA analysis (K) and the correlation between each module and the age-related shape (L)(Student's t-test, ^*^*q* < 0.05,^**^*q* < 0.01,^***^*q* < 0.001,^****^*q* < 0.0001).


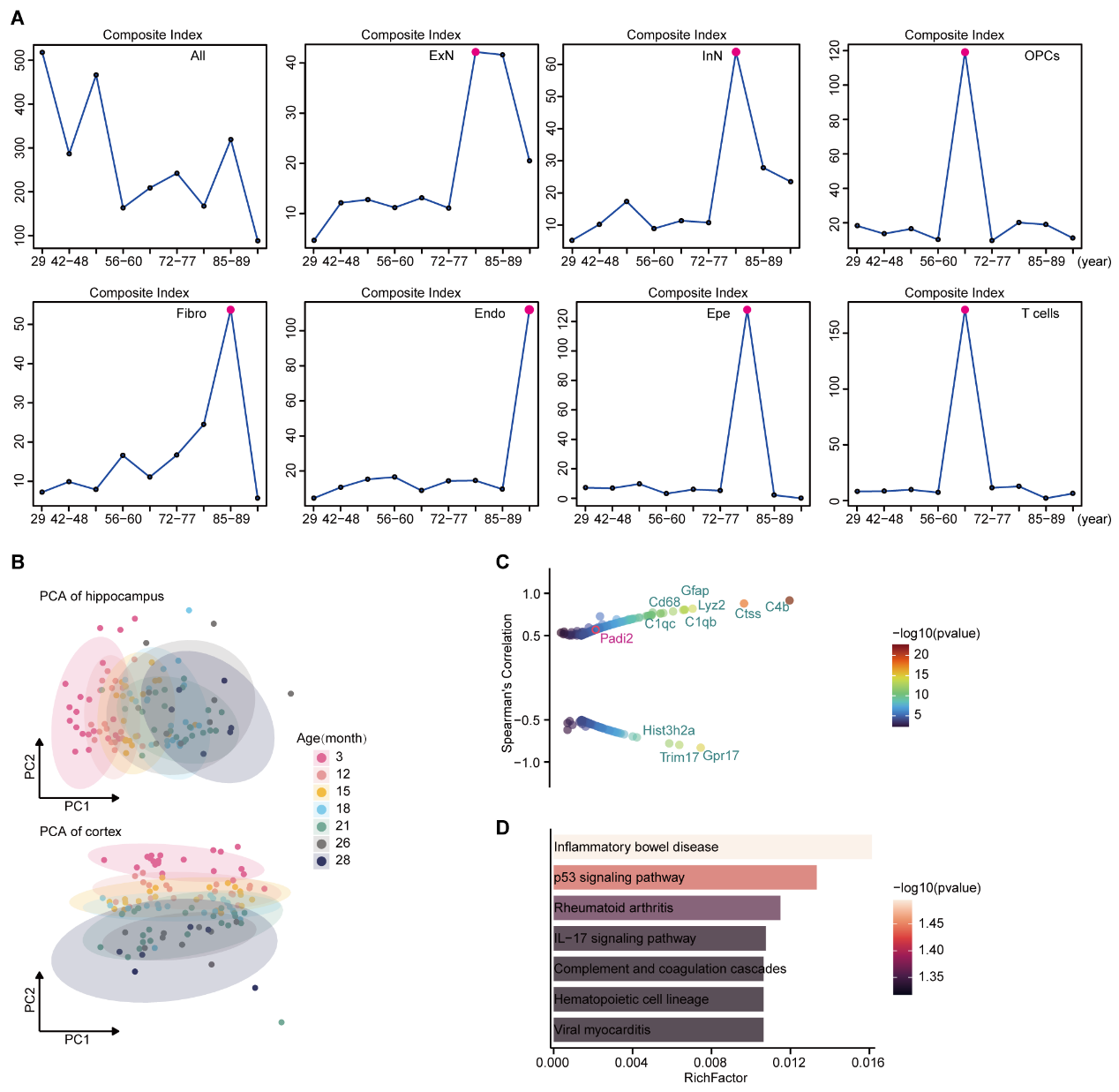


**Figure S9.** Tipping point timing similar to humans in healthy aging brains of mice. A) Age-related tipping points overall and in each cell type. B) The PCA results show the distribution of age samples in the published mouse data. C) Age-related genes in the mouse hippocampus. D) The KEGG pathway enrichment of key DNB molecules at tipping point in mice.
