# Supplementary figures and images for: Identifying Tipping Points during Healthy Brain Aging through Single‐Nucleus Transcriptomic Analysis
Source: Adv Sci (Weinh). 2025 Aug 19;12(41):e05779. doi: 10.1002/advs.202505779 (PMC12591157; doi:10.1002/advs.202505779)

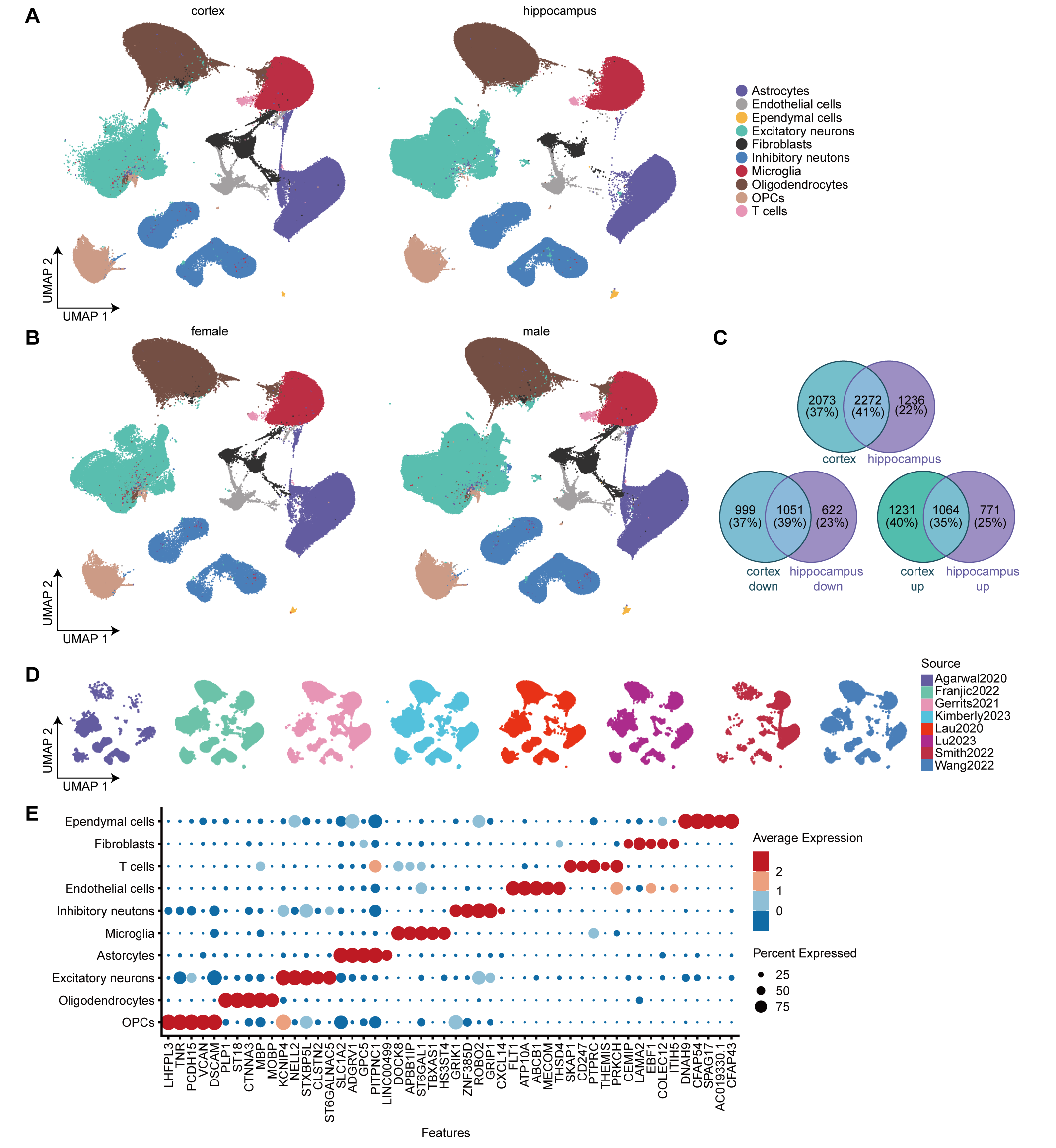

Supplement: Supplementary file 9 — Supporting Information [file ADVS-12-e05779-s006.zip › Supplement Fig1.tif]

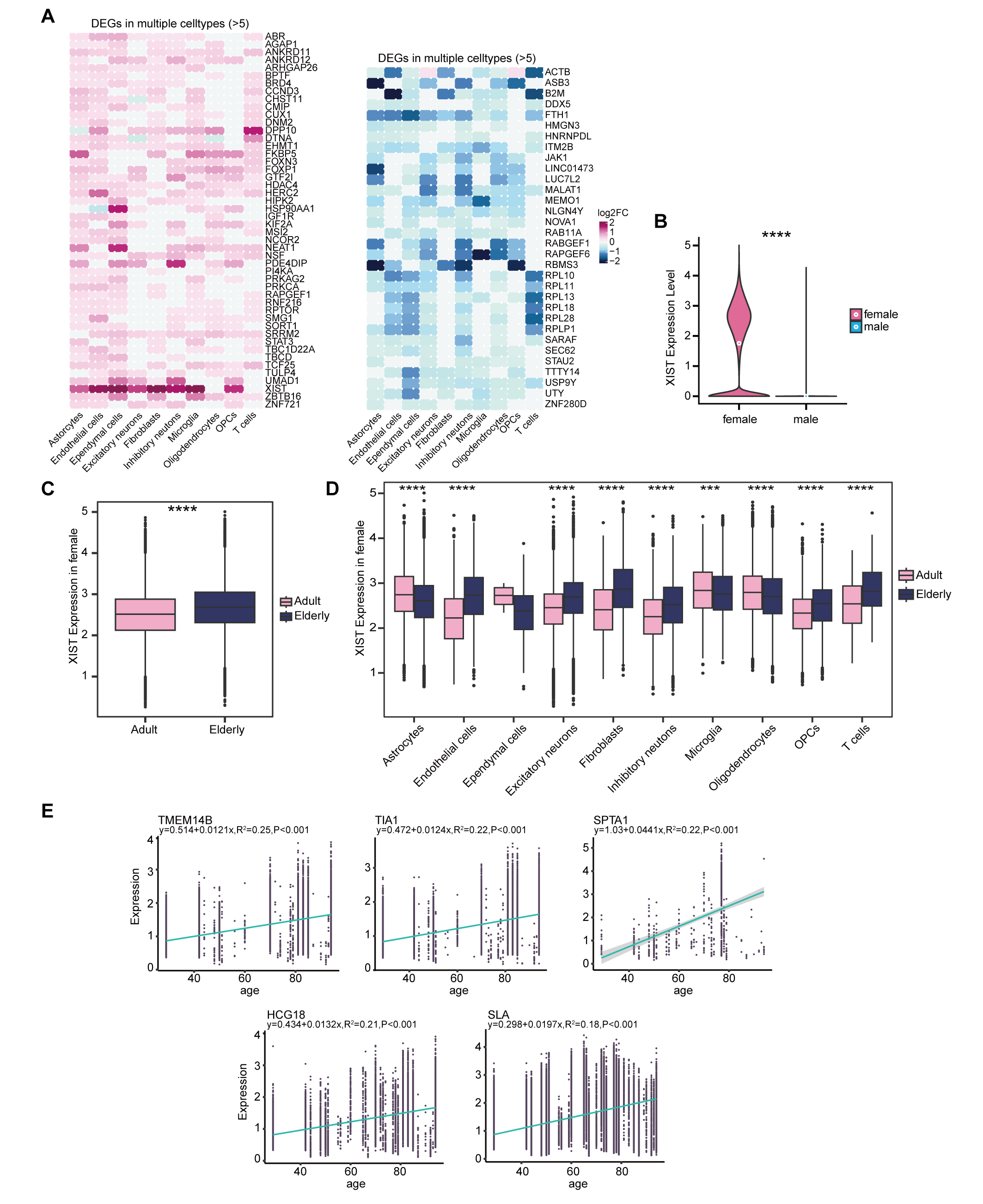

Supplement: Supplementary file 9 — Supporting Information [file ADVS-12-e05779-s006.zip › Supplement Fig2.tif]

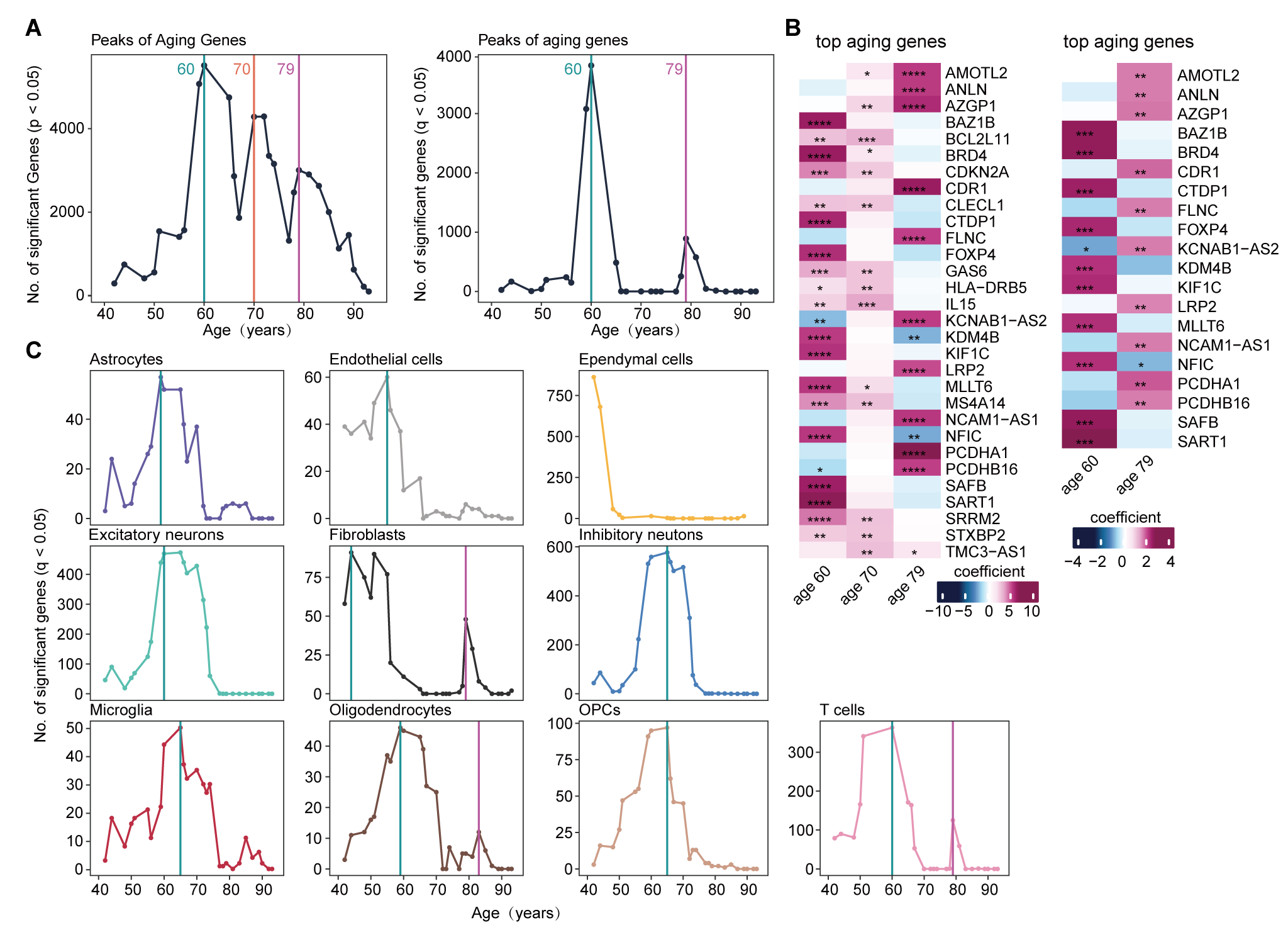

Supplement: Supplementary file 9 — Supporting Information [file ADVS-12-e05779-s006.zip › Supplement Fig3.tif]

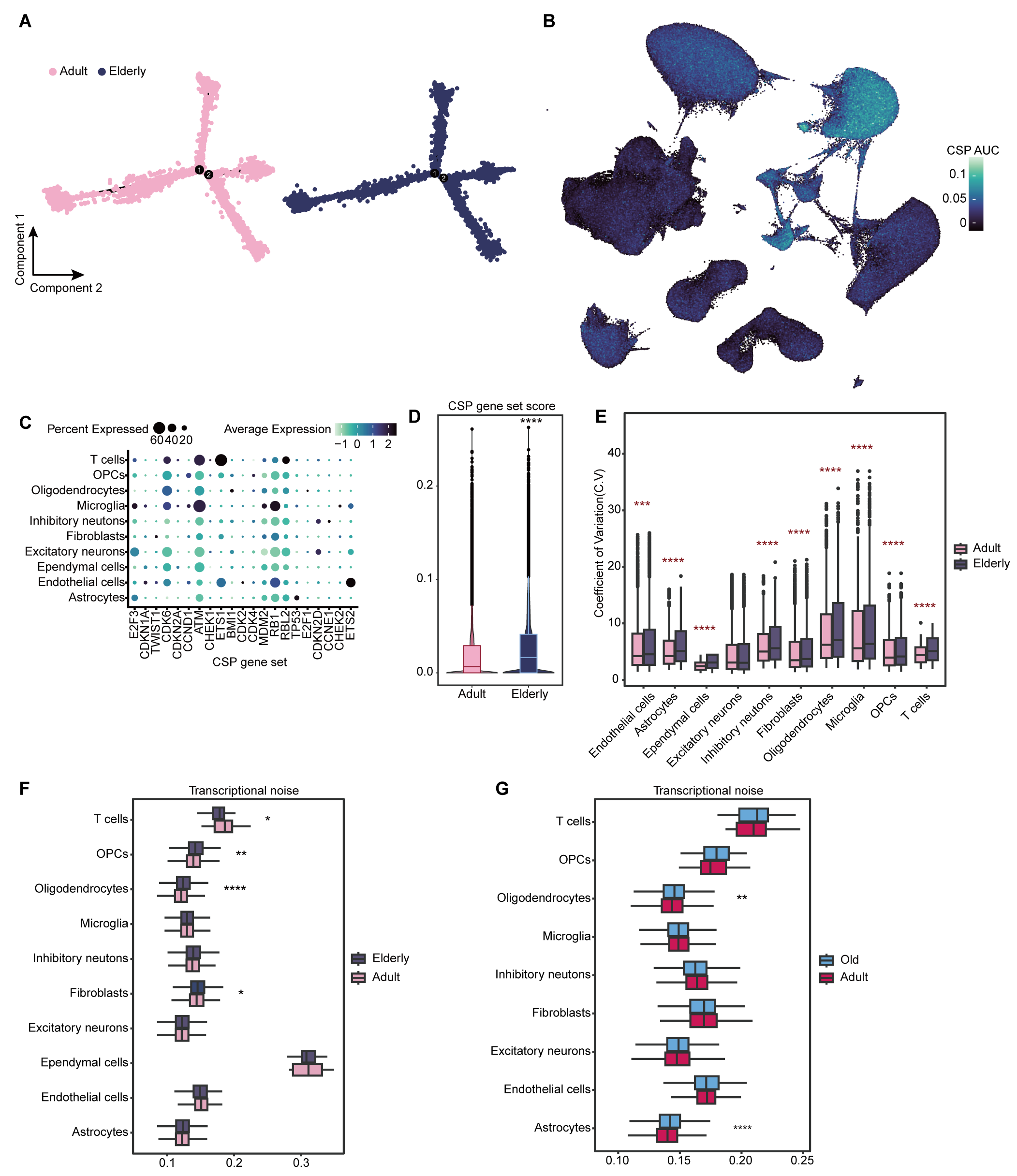

Supplement: Supplementary file 9 — Supporting Information [file ADVS-12-e05779-s006.zip › Supplement Fig4.tif]

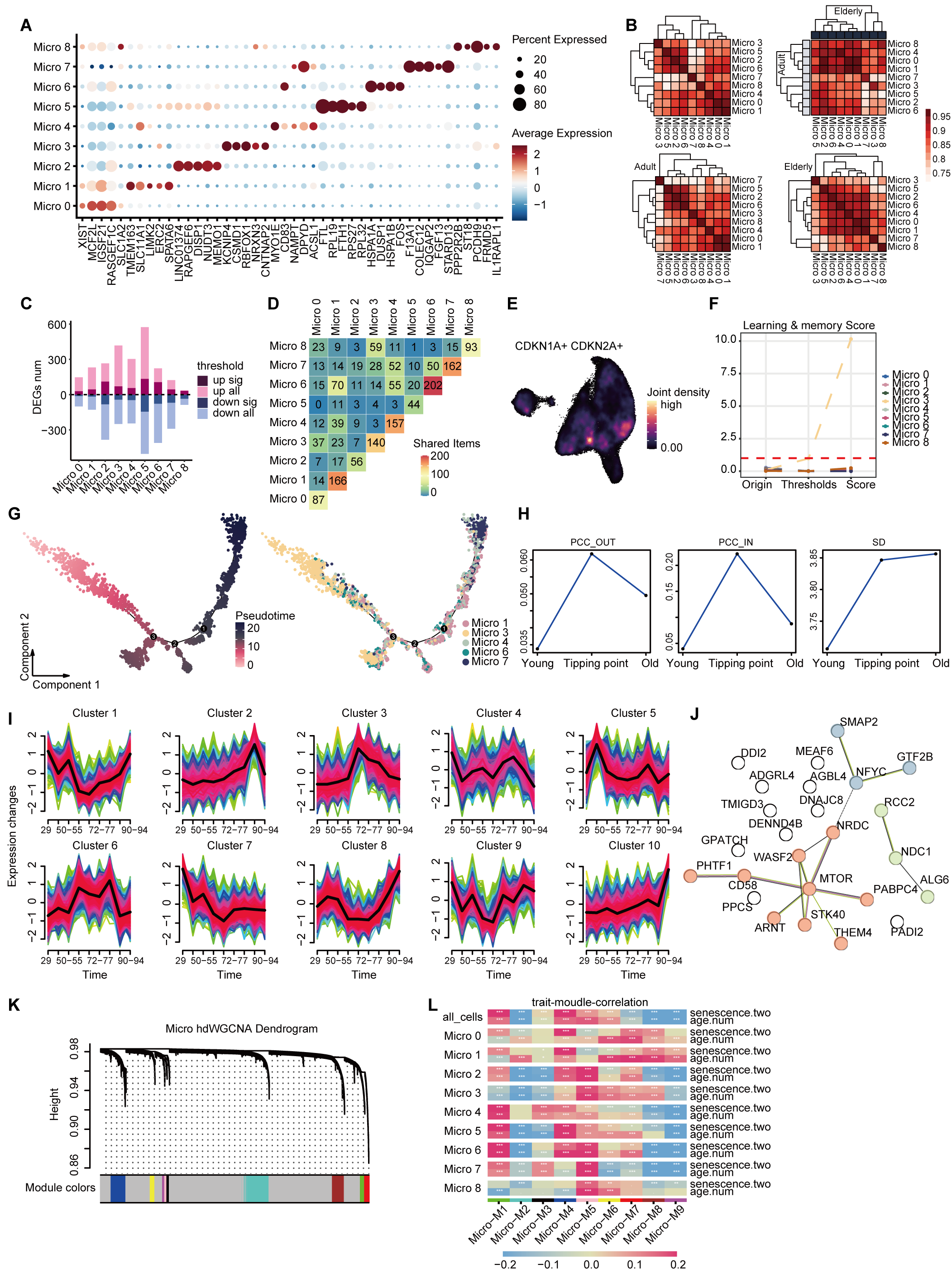

Supplement: Supplementary file 9 — Supporting Information [file ADVS-12-e05779-s006.zip › Supplement Fig5.tif]

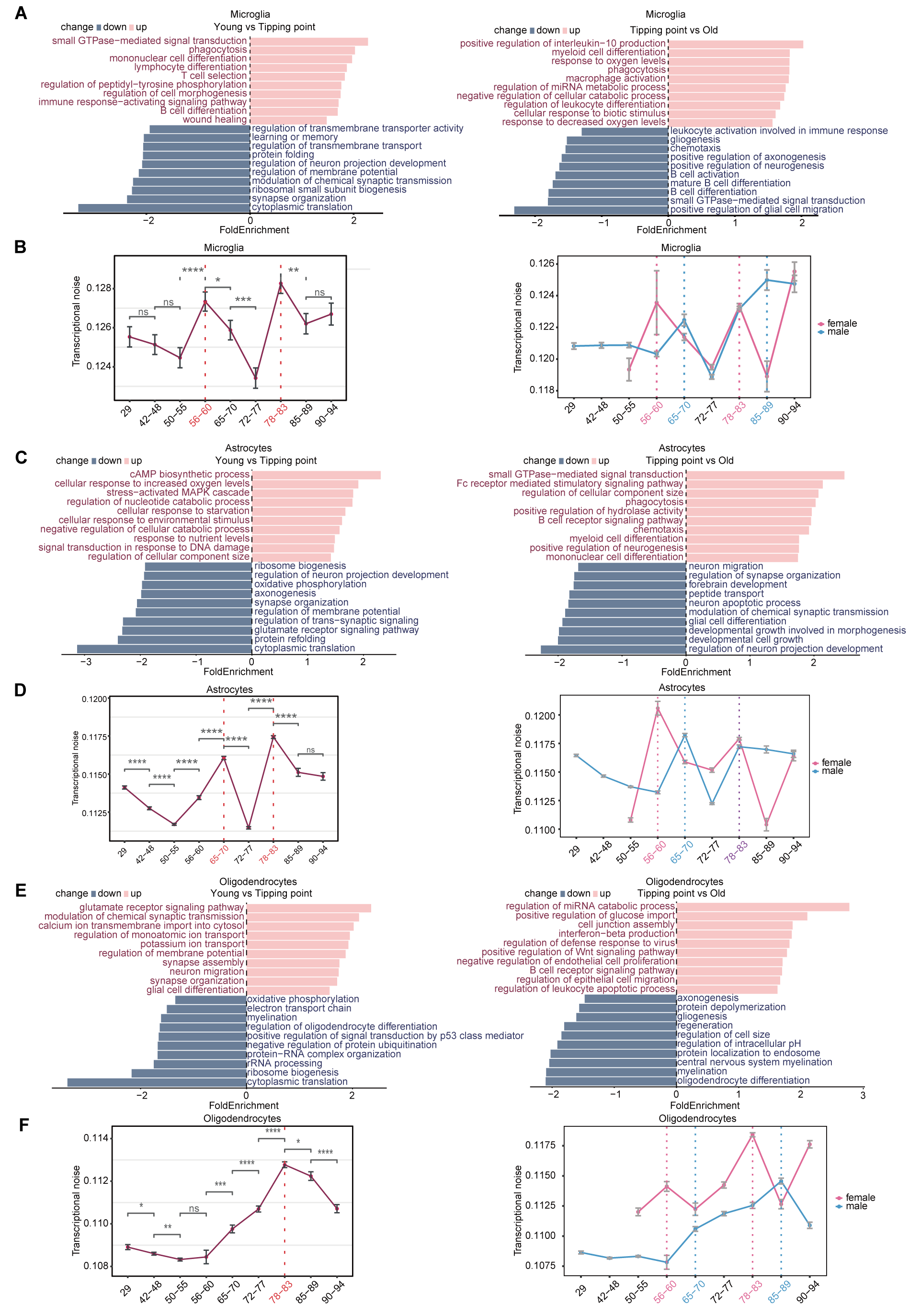

Supplement: Supplementary file 9 — Supporting Information [file ADVS-12-e05779-s006.zip › Supplement Fig6.tif]

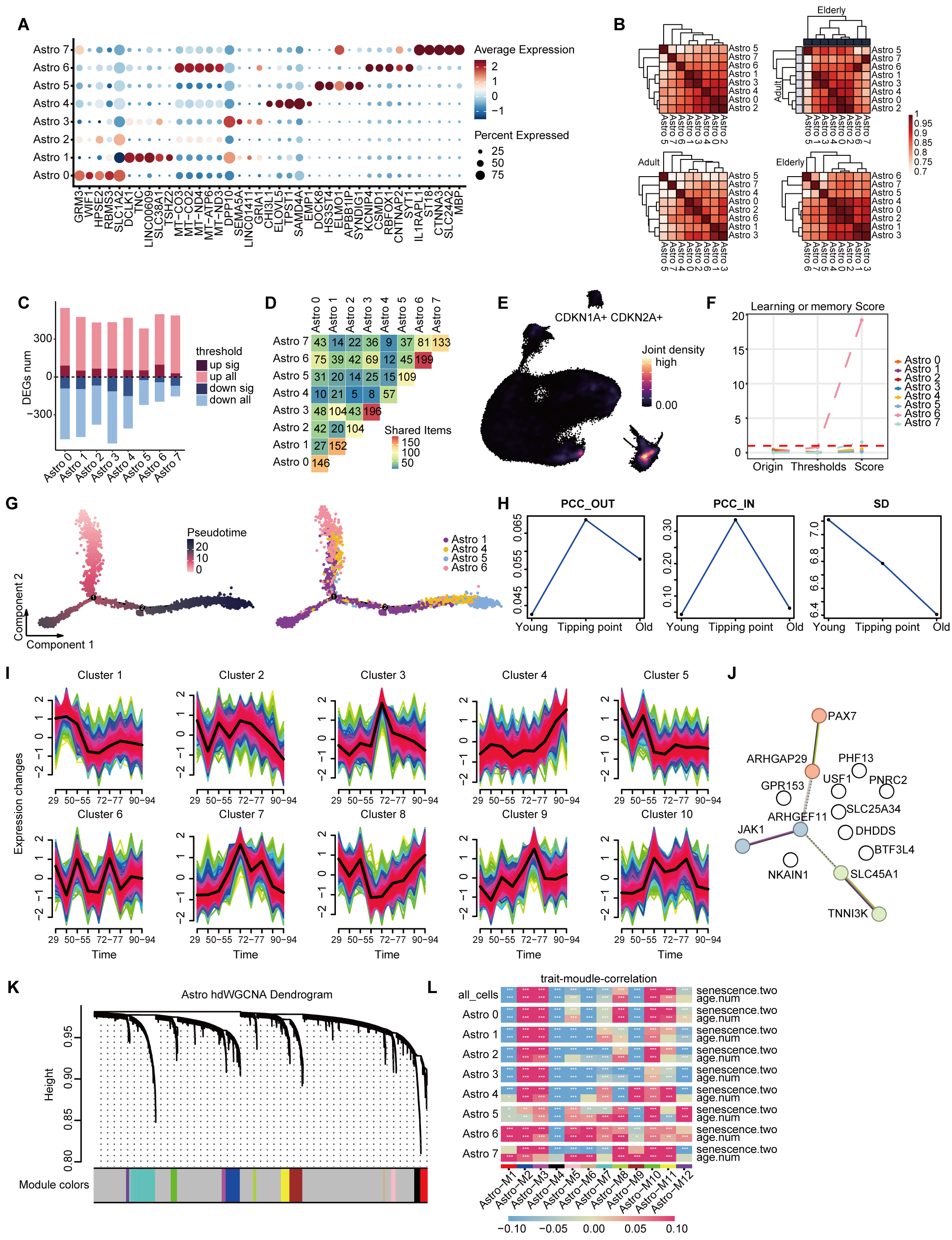

Supplement: Supplementary file 9 — Supporting Information [file ADVS-12-e05779-s006.zip › Supplement Fig7.tif]

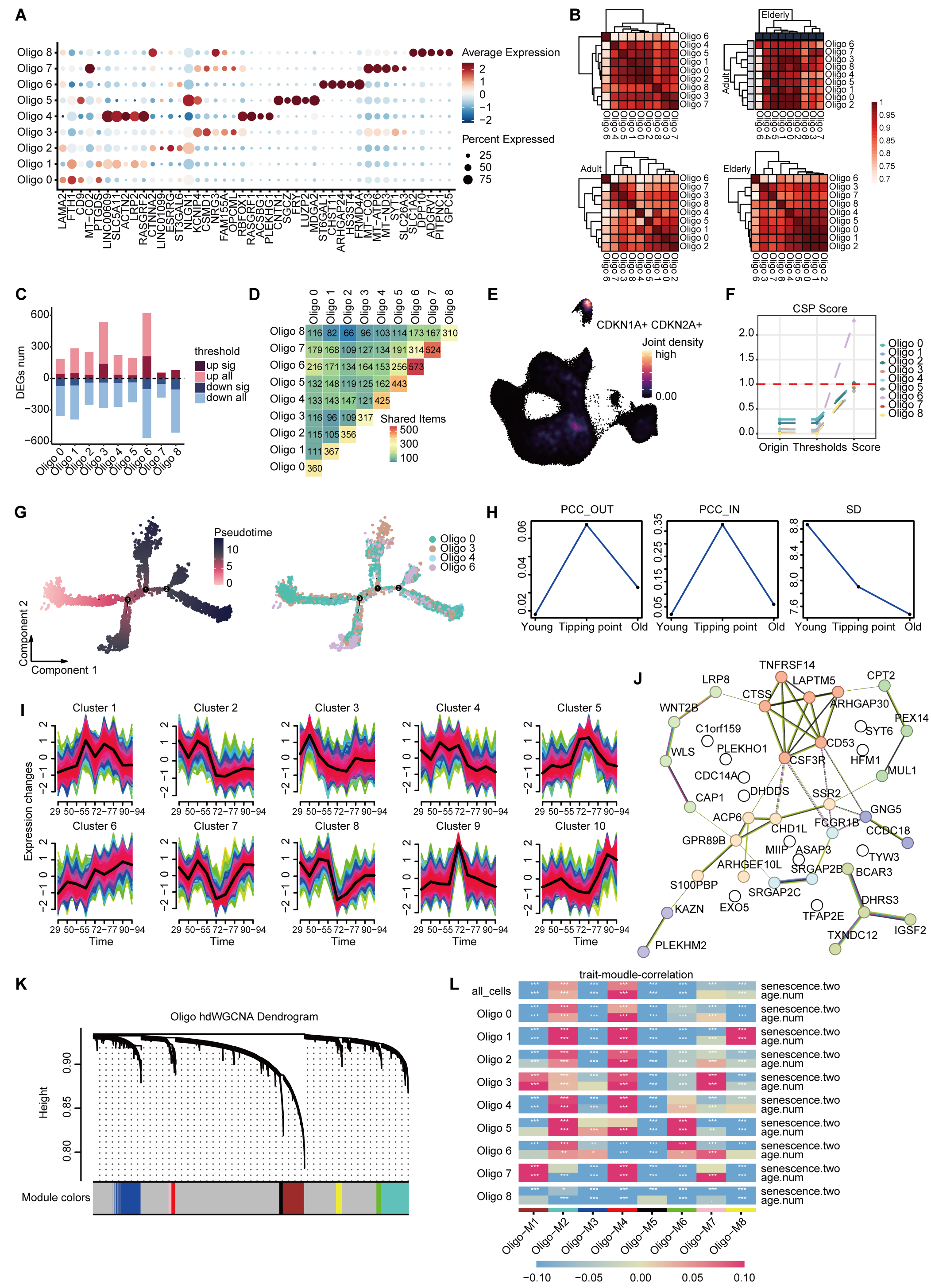

Supplement: Supplementary file 9 — Supporting Information [file ADVS-12-e05779-s006.zip › Supplement Fig8.tif]

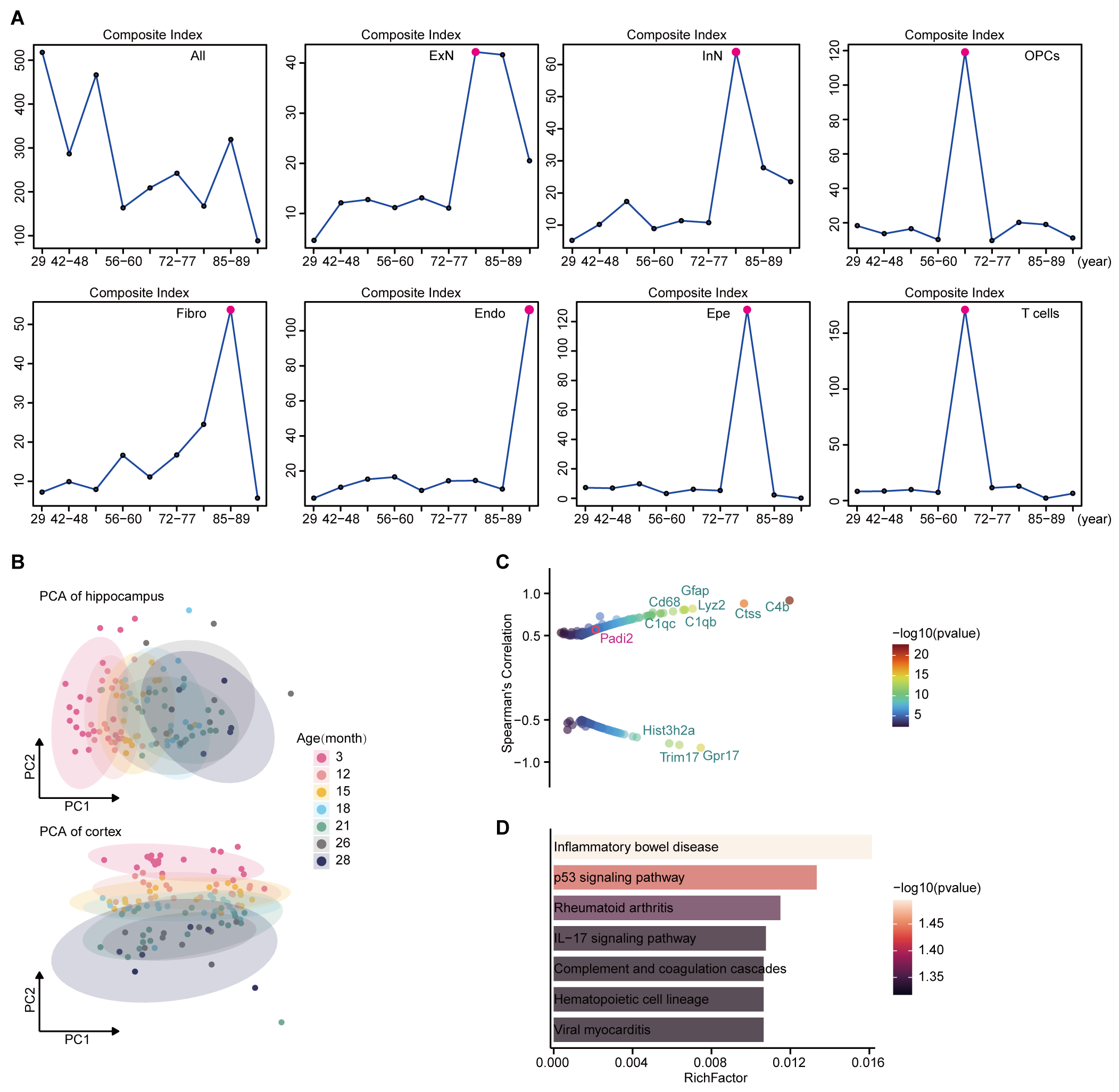

Supplement: Supplementary file 9 — Supporting Information [file ADVS-12-e05779-s006.zip › Supplement Fig9.tif]
